# Neuroplastic Responses to Chiropractic Care: Broad Impacts on Pain, Mood, Sleep, and Quality of Life

**DOI:** 10.3390/brainsci14111124

**Published:** 2024-11-07

**Authors:** Heidi Haavik, Imran Khan Niazi, Imran Amjad, Nitika Kumari, Usman Ghani, Moeez Ashfaque, Usman Rashid, Muhammad Samran Navid, Ernest Nlandu Kamavuako, Amit N. Pujari, Kelly Holt

**Affiliations:** 1 Centre for Chiropractic Research, New Zealand College of Chiropractic, Auckland 1060, New Zealand; imran.amjad@nzchiro.co.nz (I.A.); nitika.kumari@nzchiro.co.nz (N.K.); usman.ghani@nzchiro.co.nz (U.G.); usman.rashid@nzchiro.co.nz (U.R.); kelly.holt@nzchiro.co.nz (K.H.); 2 Faculty of Health & Environmental Sciences, Health & Rehabilitation Research Institute, Auckland University of Technology, Auckland 1010, New Zealand; 3 Centre for Sensory-Motor Interactions, Department of Health Science and Technology, Aalborg University, 9220 Aalborg, Denmark; 4 Faculty of Rehabilitation and Allied Health Sciences, Riphah International University, Islamabad 46000, Pakistan; 5 School of Physics, Engineering and Computer Science, University of Hertfordshire, Hatfield AL10 9AB, UK; m.ashfaque@herts.ac.uk (M.A.); amit.pujari@ieee.org (A.N.P.); 6 Donders Institute for Brain, Cognition and Behaviour, Radboud University Medical Center, 6525 Nijmegen, The Netherlands; samran.navid@nzchiro.co.nz; 7 Centre for Robotics Research, Department of Informatics, King’s College, London WC2G 4BG, UK; ernest.kamavuako@kcl.ac.uk; 8 School of Engineering, University of Aberdeen, Aberdeen AB24 3FX, UK

**Keywords:** chiropractic, electroencephalogram (EEG), somatosensory evoked potentials (SEPs), default mode network (DMN), PROMIS-29

## Abstract

Objectives: This study aimed to elucidate the mechanisms of chiropractic care using resting electroencephalography (EEG), somatosensory evoked potentials (SEPs), clinical health assessments (Fitbit), and Patient-reported Outcomes Measurement Information System (PROMIS-29). Methods: Seventy-six people with chronic low back pain (mean age ± SD: 45 ± 11 years, 33 female) were randomised into control (*n* = 38) and chiropractic (*n* = 38) groups. EEG and SEPs were collected pre and post the first intervention and post 4 weeks of intervention. PROMIS-29 was measured pre and post 4 weeks. Fitbit data were recorded continuously. Results: Spectral analysis of resting EEG showed a significant increase in Theta, Alpha and Beta, and a significant decrease in Delta power in the chiropractic group post intervention. Source localisation revealed a significant increase in Alpha activity within the Default Mode Network (DMN) post intervention and post 4 weeks. A significant decrease in N30 SEP peak amplitude post intervention and post 4 weeks was found in the chiropractic group. Source localisation demonstrated significant changes in Alpha and Beta power within the DMN post-intervention and post 4 weeks. Significant improvements in light sleep stage were observed in the chiropractic group along with enhanced overall quality of life post 4 weeks, including significant reductions in anxiety, depression, fatigue, and pain. Conclusions: These findings indicate that many health benefits of chiropractic care are due to altered brain activity.

## 1. Introduction

Chiropractic care is based on the premise that correcting vertebral subluxations improves central neural function, improving human performance and clinical outcomes [1,2,3]. Vertebral subluxation is recognised by the World Health Organization as a biomechanical lesion within the vertebral column and is classified under the ICD-10-CM code M99.1 [4]. It is characterised by abnormal movement or function of spinal segments which is identified by clinical markers such as restricted intersegmental range of motion, tenderness upon palpation, palpable asymmetric intervertebral muscle tension, and altered joint play and end feel [5,6]. Vertebral subluxation involves altered afferent input from muscle spindles in the paraspinal muscles at the subluxated levels [2,7]. This disruption in sensory input can cause maladaptive central neural plastic changes, resulting in impaired sensorimotor integration and control [2,8,9,10]. These maladaptive central plastic changes in the central nervous system (CNS) can worsen over time [2,7,11,12,13], which is thought to contribute to dysfunction, pain, and other symptoms by disrupting normal sensorimotor control [2,7]. These maladaptive central plastic changes are thought to be reversed or improved through chiropractic care [2,12,13,14].

Over the past decade, multiple studies have shown that chiropractic adjustments alter central neural function and improve the ability of peripheral muscles to produce force [2,15,16,17,18,19,20]. Chiropractic adjustments have been shown to influence various aspects of neurophysiology, including somatosensory processing, sensorimotor integration, and motor control [1,2,18,19,21,22,23], all crucial for executing motor tasks accurately and recovering from central nervous system injuries [24,25]. Notably, a single session of chiropractic spinal adjustment was found to increase plantar flexor muscle strength in college students [18], in elite athletes [15] and in stroke survivors [20]. Moreover, when chiropractic spinal adjustments were combined with physical therapy over four weeks in people with chronic stroke, it led to greater improvements in motor function compared to physical therapy alone or physical therapy combined with sham chiropractic care [26]. Despite these promising findings, it is still unclear how neurophysiological alterations after chiropractic care translate into clinically significant improvements in function and overall quality of life; i.e., the mechanisms involved are not yet fully elucidated.

Work to date, using techniques such as transcranial magnetic stimulation and somatosensory evoked potentials (SEPs), have studied the effects of chiropractic care and found that neuroplastic brain changes occur in structures such as the primary somatosensory cortex, primary motor cortex, prefrontal cortex (PFC), and cerebellum [16,17,27,28]. Improvements in either prefrontal cortex or cerebellar function could explain many of the clinically relevant changes that have been documented following chiropractic care, such as improved joint position sense error [29], cortical processing [17,30], reflex excitability [31], reaction time [30], cortical sensorimotor integration [17,28], motor control [2], upper and lower limb muscle functions [2,15,18,20,32,33,34,35], and pain changes, particularly changes in unpleasantness feelings and catastrophising [36]. The PFC is considered the central hub for mental comorbidities associated with chronic pain, including not only pain unpleasantness feelings but also depressive mood, impaired cognition, and pain catastrophising, among others [36]. Both the PFC and the Cerebellum are fundamental structures responsible for multimodal integration and an accurate inner body schema and external world schema [37,38,39]. It is also becoming increasingly evident that both the PFC and cerebellum influence emotional control and mental health [40,41,42], as well as neuroendocrine responses [43,44], autonomic nervous system function [45,46], and immune function [47,48].

To examine the neuroplastic alterations that occur following chiropractic adjustments, neurophysiological measures such as functional magnetic resonance imaging (fMRI) and electroencephalography (EEG) can be used. More recently, the literature has begun to emphasise the importance of synchronised activities across brain regions, which can be measured using functional connectivity analysis [49]. Connectivity analysis is a powerful method for examining the communication and collaboration between different brain regions, i.e., networks within the brain [50]. It involves measuring functional connectivity (communication between brain sources or sensors), including linear and non-linear processes within the brain [51]. Using fMRI, Gay and colleagues [52] found changes in resting functional connectivity within the pain processing network (PPN) and decreased pain intensity after manual therapy in people with experimentally induced muscle-related low back pain. Even though functional connectivity can be analysed directly from fMRI measurements, EEG is considered a superior method to study functional connectivity as it provides better temporal resolution, accessibility, and lower examination costs [53,54].

With EEG, functional connectivity analysis can be performed at the sensor level [55] or the source level [56]. EEG signals at the sensor level express a complex mixture of overlapping signals from other EEG sensors due to volume conduction [57]. Therefore, EEG source reconstruction is considered more robust for calculating functional connectivity between sources [56]. Irrespective of sensor and source-based approaches, two widely used approaches to measure functional connectivity are (1) Coherence and (2) Phase lag index (PLI). Coherence, a commonly used method, evaluates the linear relationships between signals by measuring the consistency of phase variances between two signals over time [58]. In contrast, PLI evaluates non-linear relationships by quantifying the asymmetry of the distribution of phase variances between signals. It focuses on genuine interactions and avoids spurious correlations caused by volume conduction effects [58]. Therefore, PLI is the preferred method to measure functional connectivity.

Two previous studies have explored EEG connectivity changes before and after chiropractic care using PLI at source level [59,60]. One of these studies explored the effects of chiropractic care on tonic pain [59], and the other explored the effects of chiropractic care in stroke survivors [60]. In the first study, a single session of chiropractic adjustments appeared to alter the way the brain processes tonic pain signals [59]. In the stroke survivors, there was a significant increase in functional connectivity in the Alpha band within the default mode network (DMN), in particular, an increase in functional connectivity between the posterior cingulate cortex (PCC) and parahippocampal (ParaH) regions [60]. However, these findings have only been examined in stroke survivors to date.

Therefore, this study aimed to investigate the immediate and prolonged effect of chiropractic care on various neurophysiological (resting state EEG and SEPs) and clinical health parameters (using a Fitbit and patient-reported outcome measure (PROM)) in people with non-specific chronic low back pain (CLBP), to better understand the mechanisms of chiropractic care.

## 2. Materials and Methods

### 2.1. Design and Setting

This parallel-group randomised controlled design study was conducted at a private chiropractic clinic in Henley-on-Thames, England, in 2022/2023. The study was approved by the Research Ethics Panel of King’s College London (HR-20/21-20326, 2 November 2020). The New Zealand College Chiropractic Research Committee also approved the study. The study was conducted in accordance with the Declaration of Helsinki.

### 2.2. Participants

Seventy-six people with non-specific CLBP participated in the study. They were recruited from the surrounding areas of Henley-on-Thames, England, through local advertising. Participants were divided into the following two groups: an intervention + usual care group (*n* = 38) and a usual care control group (*n* = 38). Participants were included if they were aged between 18 and 60 years, had pain located between the lower rib margins and the buttock crease [61] present for at least three months in the last year to be counted as CLBP [62], and had no identifiable patho-anatomical cause for the pain [63]. Participants were excluded from the study if their current pain was above 7/10 on a visual analogue pain scale, had a history of previous fractures, previous spinal surgery, high blood pressure, and metabolic, inflammatory, or neoplastic disease, or they were unable to perform the assessment procedures due to contraindications or movement limitations. All participants gave their written informed consent prior to participating in the study.

### 2.3. Procedure

Following recruitment and screening, eligible participants were randomly divided to receive either four weeks of usual care (control) or four weeks chiropractic + usual care (intervention) using a computer-generated block randomisation scheme (5 blocks). The EEG data (resting + SEPs) was measured before (pre-intervention), immediately after the first session (post intervention), and after 4 weeks of intervention or control (post 4 weeks intervention) by an investigator with over 10 years of experience in the field. The PROMIS-29 was completed before and after the 4 weeks of intervention. Sleep stages and physical activity were continuously recorded by Fitbit watches for four weeks in both groups. Blinding the chiropractor or participants to intervention assignment was not feasible due to the manual nature of the intervention. Nonetheless, all essential study investigators and data analysts remained unaware of the intervention group allocation details throughout the study period and until the final analysis was concluded. All recorded data underwent anonymisation and were assigned a pre-established code to conceal group allocation and ensure appropriate blinding.

### 2.4. Interventions

The chiropractic care and usual care control interventions were similar to those used in previous studies that have investigated the neurophysiological effects of chiropractic spinal adjustments [1,2,20,26,60,64,65,66].

#### 2.4.1. Chiropractic Care

The participants in this group underwent usual care in addition to four weeks of the chiropractic care that included manual high-velocity low-amplitude (HVLA) adjustments to the spine or pelvic joints (pubic symphysis, sacroiliac joints, and sacrococcygeal joints) identified as being subluxated [6,67]. These HVLA directed at vertebral subluxations rapidly stretch the surrounding paraspinal tissues and, in particular, the deep small paraspinal muscles [68,69,70,71,72,73,74]. This results in a “bombardment” of proprioceptive input to the CNS that elicits the changes in central neural excitability and motor control changes [2,68,69,70,71,72,73,74,75,76,77,78]. The sites selected for spinal adjustment were based on the chiropractors experience and included clinical indicators of vertebral subluxations [6], such as tenderness to palpation of the relevant joints, manual palpation for restricted intersegmental range of movement, palpable asymmetric intervertebral muscle tension, and any unusual or blocked joint play and end feel of the joints [5,6]. Chiropractors routinely use these biomechanical and neurophysiological markers to identify vertebral subluxations and as clinical indicators for chiropractic care [5,6]. Multiple levels of the spine were adjusted in each participant if required. Chiropractic care was provided approximately three times per week for four weeks, with each visit lasting about 15 min. The chiropractor who performed all the experimental interventions and the first control intervention session was a highly accomplished practitioner with 30 years of clinical experience. He is trained in multiple different chiropractic techniques and has created his own system for pragmatically determining what each patient needs clinically.

#### 2.4.2. Usual Care (Control)

Usual care referred to any care recommended or prescribed by non-chiropractic health providers for CLBP, including self-management advice, pharmacologic pain management, physical therapy, or referral to a pain clinic. Along with usual care, participants underwent the same assessment for spinal and pelvic dysfunction as the chiropractic care group for the first control session only. This group’s first control session, therefore, acted as a physiological control for possible changes occurring due to the cutaneous, muscular, or vestibular input that would have occurred with the passive and active movements involved in preparing a patient for chiropractic spinal adjustments. Thus, the only difference between the first intervention and the first usual care control is the application of the HVLA thrusts to the dysfunctional spinal segments. During this first control session, this group, therefore, acted as a control for the time it takes to perform the HVLA adjustments, and the touch and movement of the participant that occurs as the chiropractor moves them into an adjustment setup. During the adjustment setups for this first control intervention, the chiropractor was careful not to thrust on the spine or take a vertebral segment to end-range tension. The duration of the first control intervention was similar to that of the chiropractic care. For the rest of the four-week control period, the control group had no interaction with the chiropractor or researchers, carrying on with any recommendations prescribed by non-chiropractic health providers for their CLBP.

### 2.5. Outcome Measures

#### 2.5.1. Electroencephalogram (EEG)

EEG was recorded using a 64 channel Brainwave EEG cap coupled with a REFA amplifier (TMSi, Oldenzaal, The Netherlands) at a sampling rate of 2.048 Hz. The recordings were obtained from 64 scalp sites, following the 10–20 electrode system [79], with the ground electrode situated at AFz and both mastoids (M1 and M2) employed as reference points. Electrode impedance was consistently maintained below 10 kΩ. Participants were seated comfortably in a chair, in front of a screen, and were asked to focus on a white fixation cross with black background displayed in the centre of a computer screen while minimising eye blinks, eye movements, and facial movements for three minutes (resting state EEG).

#### 2.5.2. Somatosensory Evoked Potentials (SEPs)

The median nerve was stimulated using electrical pulses delivered by the electrical stimulator (Digitimer DS7AH, Welwyn Garden City, UK) to evoke SEPs, as done in our previous studies from our research group [17,22,80,81]. The stimulation electrodes (Neuroline 700, AMBU A/S, Ballerup, Denmark) were placed at the left wrist. The motor threshold was defined as the lowest current intensity, which elicited a visible twitch of the thumb. Before and after chiropractic care or control, 1000 electrical pulses were given to the median nerve. The stimulation pulse was monophasic, with a width of 0.2 ms and a frequency of 2.3 Hz.

#### 2.5.3. Sleep and Physical Activity Using Fitbit

The use of commercially available wearable devices that monitor physiological activity, such as heart rate, number of steps taken, and sleep stages, is growing [82]. These wearable devices are an easy and non-invasive method for capturing a wide range of physiological measures from the wearer [82]. Using the Fitabase analytics system (Small Steps Labs, San Diego, CA, USA), data from physical activity trackers were remotely collected and aggregated whenever data were transmitted to the users’ personal Fitbit dashboards. Data captured included time in different sleep stages (awake, light, deep, rapid eye movement (REM)) per 30 s, time spent on different levels of physical activity (lightly active, fairly active, very active) per day, and heart rate per 5 s (the lowest resolution available using the Fitabase platform [83].

#### 2.5.4. Patient-Reported Outcome Measure

Patient-reported Outcomes Measurement Information System (PROMIS-29 v2.0) was used to measure Health-related quality of life (QOL). It assesses pain intensity using a single 0–10 numeric rating item and seven health domains (physical function, fatigue, pain interference, depressive symptoms, anxiety, ability to participate in social roles and activities, and sleep disturbance), using four items per domain [84]. These comorbidities are highly correlated with CLBP [85,86]. The total score for all questions was calculated by summing individual scores, and subscores for each domain were also calculated. The PROMIS-29 v2.0 is a reliable and valid instrument that can be used to assess the impacts of health care and track changes in health over time [84].

### 2.6. Data Analysis

#### 2.6.1. EEG Data Preprocessing

**Figure 1:**
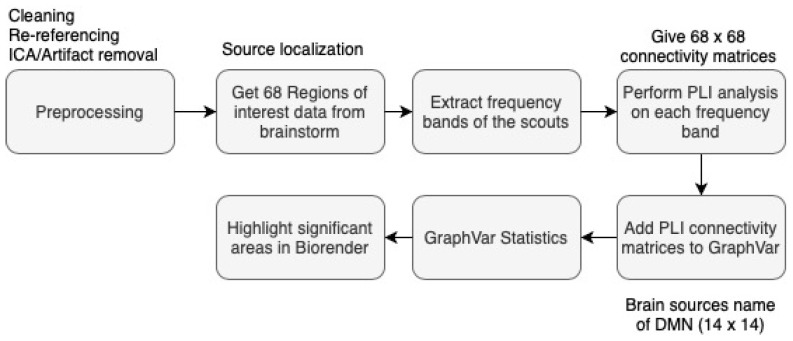
Data Analysis Pipeline for EEG.

The overall flow graph of the EEG data processing for the study is shown in Figure 1.

The raw EEG data were preprocessed offline using EEGLAB (version 14.1.1) [87] and ERPLAB (version 6.1.4) [88] running on MATLAB (2015b) (The MathWorks, Inc., Natick, MA, USA).

For the EEG preprocessing, 62 electrodes were re-referenced to achieve a common average reference excluding the two mastoids. PREP pipeline (version 0.55.1) [89] was used to remove and interpolate bad channels and remove line noise. After running the PREP pipeline, epochs were extracted for SEPs from −20 to 300 ms to the stimulus and were baseline-corrected using the pre-stimulus period. The bad epochs were visually removed from the data before running the independent component analysis (ICA). Custom-written ICA code was then used to remove bad components from the SEPs and continuous EEG data. The SEP data obtained after ICA were subjected to the ERPLAB artifact detection algorithm of moving window threshold [88]. A 20 ms window width and a 5 ms step were defined with a threshold of ±100 μV. The epochs in which the signal exceeded ±100 μV on any channel were rejected. This preprocessed data were then used for multiple analyses, including (1) resting-state EEG spectral analysis, as well as (2) resting-state EEG and SEPs source localisation and functional connectivity analysis.

#### 2.6.2. Resting EEG Spectral Analysis

The EEG power spectral analysis used FieldTrip toolbox (version 20180912). To estimate the differences in the power spectrum of the five EEG frequency bands between the conditions mentioned in the section above, the power spectra between 1 and 32 Hz of the resting state EEG were calculated using a Fourier basis with a Hanning window of 1 s, which was followed by computation of the average power of each frequency band. Difference plots of each frequency band were used across two sessions at a time (for example, Post vs. Pre in the chiro–control group), and so on. This comparison identifies any changes between the two groups.

#### 2.6.3. EEG Source Localisation Analysis

##### EEG Source Reconstruction

**Figure 2:**
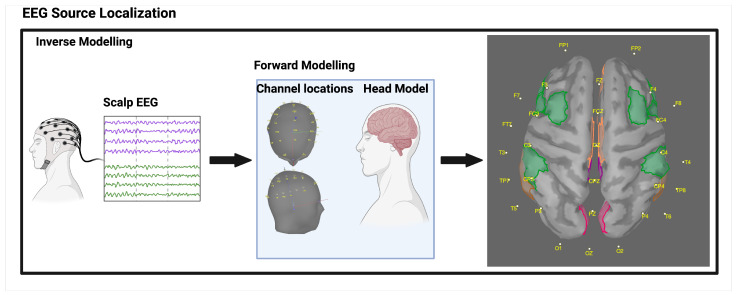
Source localisation (Forward and inverse modelling).

Volume conduction at the sensor level of EEG makes it inappropriate to analyse the functional connectivity [57]. Furthermore, the sensor signals are a complex mixture of signals from multiple brain regions overlapping each other [56]. Therefore, functional connectivity does not represent activity within the brain well. In this study, the concept of source localisation was used to avoid issues associated with volume conduction. EEG source reconstruction was performed using Brainstorm in MATLAB R2022a [90]. Source reconstruction is a method used in neuroscience to estimate the location and activity of underlying neural sources based on measurements obtained from multiple sensors or electrodes [91]. Using advanced mathematical models and algorithms, source reconstruction can provide a more comprehensive understanding of brain activity by identifying the specific regions and networks involved in specific tasks [92]. There are two main steps in EEG source reconstruction: forward modelling and inverse modelling, both dependent on each other for accurate source reconstruction [93]. Forward modelling involves the human head, including its scalp, skull, cortex, and electromagnetic properties [56]. The inverse modelling uses the information about cortical activity from forward modelling to identify the most likely locations and strengths of brain activity [93]. Both forward and inverse modelling are highlighted in Figure 2.

##### Forward Modelling

This section outlines the forward modelling process for EEG source reconstruction. The goal was to determine the location and orientation of EEG sensors relative to the cortical source, which required defining the location and orientation of current dipoles [90,93]. This was accomplished by placing source dipoles on a voxel grid space approximating the cortical space, ensuring they were oriented perpendicular to the cortex. Symmetric boundary element method (Open MEEG BEM) was used to model the dipoles [90]. A default generic head model from Brainstorm May 2019, which featured 15,000 vertices and a three-layer compartment (scalp, skull, and brain), was employed. Tissue conductivities were set based on a previous study by Sadleir and Argibay [93]: scalp = 1, skull = 0.0125, and brain = 1. The forward model was calculated after defining the 64 electrode locations (including ref: M1 and M2) on the scalp using the 10–20 electrode placement system and the Colin27 Neuroscan Quick-Cap 64.

##### Inverse Modelling

The inverse problem is a technique used in neuroscience to estimate activity from the brain based on measurements taken from EEG sensors [94]. This technique solves an underdetermined and ill-posed problem where the number of estimated sources exceeds the number of electrodes used to record the data. To achieve a solution, the method of minimum-norm estimation is utilised, which involves applying a linear kernel to the spatial data at each point in time [95]. This technique estimates cortical current source densities by minimising the overall power of the estimated sources while using an identity matrix as the noise covariance matrix [90]. However, the minimum-norm estimate tends to locate sources in the superficial regions of the cortex, leading to inaccurate results. We, therefore, used the standardised low-resolution brain electromagnetic tomography (sLORETA) method to adjust current density maps of source dipoles, representing them as normalised current densities perpendicular to the cortex [96]. To efficiently assess functional connectivity, we grouped the high-resolution sources based on the Desikan Killiany atlas, which defines 68 regions of interest (ROIs) on the cortex surface. Averaging the time series within each ROI, we formed a [ROIs x time] matrix. As suggested by the literature, we also flipped the sign of dipoles with opposite directions before averaging to prevent activity cancellation. This approach enabled accurate estimation of brain activity and provided an understanding of how different brain regions are connected.

##### Functional Connectivity Analysis

Before the functional connectivity calculation, the DMN was derived from sources only considering brain regions within this network. We used the same brain regions as in the study from [97] listed in Table 1.

##### Phase Lag Index (PLI)

The PLI is a measure of connectivity that quantifies asymmetry based on the phase difference distribution between two signals, denoted as ‘x (*t*)’ and ‘y (*t*)’. To compute the PLI, we first calculate the average phase difference using Equation (1). This requires obtaining the signal’s phase information, which is derived from the ratio phase between the signal’s Hilbert transform and the signal itself.

(1) PLIx,y=1N∑t=1Nsign[sin⁡(ϕxt−ϕyt)]

Depending on the sign, the resulting phase difference can be either positive, negative, or zero. The phase differences are evaluated across a specific window to determine the PLI, and the calculation is performed over *N* total samples contained within that window. This process enables us to quantify the extent of connectivity and asymmetry between the signals, providing valuable insights into the functional interactions of brain regions.

In this study, we divided the data into narrow-band signals using a 4th-order Butterworth filter before PLI computation. This step allowed us to extract specific frequency bands, including the following: Alpha (7.5–12.5 Hz), Beta (12.5–30 Hz), and Gamma (30–40 Hz [98]). The computation of PLI was conducted between every reconstructed EEG source signal in a pair. The result of the computed PLI lies in the interval from zero to one, where zero indicates no connectivity and one indicates maximum connectivity between signals. After the computation of PLI between every source pair, the PLI data were stored in a connectivity matrix of dimension 14 × 14 with zeros along the diagonal, giving a symmetric square matrix corresponding to the number of sources within DMN. This resulted in three 14 × 14 connectivity matrices corresponding to pre, post, and post 4 weeks within each frequency band for every participant.

#### 2.6.4. N30 Peak Amplitude Analysis

In this study, we analysed the amplitudes and latencies of the N30 SEP peak. The P22-N30 peak amplitude was calculated from the frontal electrode, as done previously [80]. The most negative (N30) peak with respect to the stimulus were identified in the time window of 15–25 ms and 25–45 ms, respectively. Afterwards, manual inspection was performed, and the identified peaks were verified by an expert in SEP analysis. The P22-N30 peak amplitude was defined as the absolute difference in the amplitude of these peaks.

#### 2.6.5. Fitbit Data

Fitbit variables were analysed at the day level. Total wear time was used to temporally normalise the start of recording of the other variables such as heart rate across participants. The first 30 days of data for each participant were included for analysis. Sleep stage (awake, light, deep, REM) data captured per 30 s were aggregated per day by summing up the number of minutes recorded in each stage out of the 24 h per day. For intuitive communication, it was expressed as a percentage of 8 h, which is generally understood as recommended sleep duration [99]. This percentage of recommended 8 h can be taken as a proxy measure of sleep efficiency. Thus, a time of 50% per day in light sleep implies 4 h of light sleep per day out of the 24 h. Similarly, different levels of physical activity (lightly active, fairly active, very active) were also aggregated per day across participants and groups.

### 2.7. Statistical Analyses

#### 2.7.1. EEG Spectral Power Analysis

A non-parametric cluster-based permutation test was used to identify the differences in the EEG power spectrum between the pre-sessions of chiropractic and control group, the post-sessions, and the post 4 weeks sessions. The clusters were defined as two or more continuous channel-power pairs, each with *p* < 0.05 from the paired two-tailed *t*-test with respect to the conditions. The *t*-values within each cluster were added to get the cluster-level statistics, and the maximum of cluster-level statistics was used as the test statistic. A cluster was considered significant if its Monte Carlo probability for each tail exceeded the threshold of 0.025 compared to the reference distribution approximated by the Monte Carlo method with 5000 permutations.

#### 2.7.2. EEG Source Level Analysis

In this study, we employed the GraphVar-2.03a toolbox to analyse 14 × 14 PLI connectivity matrices statistically [100]. We examined the connections between each pair of nodes within the matrix. To handle the challenge of multiple comparisons, GraphVar used cluster-based permutation, which organised significant links into Graph-Components, which can be considered sub-networks. These components were measured like how clusters are identified in fMRI [101]. To assess whether the size of a graph component was non-random, we compared it to randomly generated data within GraphVar. We computed a *p*-value for each non-random component. This allowed us to pinpoint significant connectivity patterns. In the statistical section of GraphVar, we used a within-subject design, where data from subjects were collected across multiple sessions (pre, post, and post 4 weeks). GraphVar calculated the mean PLI difference between two sessions simultaneously (e.g., post–pre). Importantly, these calculations only considered significant non-random graph components. The results highlighted the brain connections where the mean PLI significantly differed between sessions, helping us identify changes in connectivity patterns over time. The same analysis was run for both the chiropractic and control groups.

#### 2.7.3. EEG N30 Peak Amplitude Analysis

The mixed effect model was used to identify the effects of the intervention on the N30 amplitude using intervention (Chiropractic and Control) and session (pre, post, and post 4 weeks) as fixed factors. The between-subject variance was estimated using random intercept in the model. The models were implemented using lme4 package version 1.1.35.5 [102] in R version 4.4.1 [103]. The pairwise comparisons were obtained using the emmeans package version 1.10.3 [104], adjusted for multiple comparisons using Tukey’s HSD.

#### 2.7.4. Fitbit Data Analysis

We imported data into the R environment for statistical computing and fitted linear mixed regression to elucidate between-group differences. To account for variation across time, we included natural splines as fixed effects at the group level and as random effects at the participant level. Participant-wise random intercepts were also included in each model. Fully saturated interaction effects for the fixed effects were included in all the models. The wear time was modelled using the Beta family with a logit link function. All other variables were modelled with the assumption of normal homoscedastic residuals. The model for time recorded in sleep stages also fitted sleep-stage-wise correlated random intercepts for participants and sleep-stage-wise residual variances. All aspects of model construction were based on Akaike’s Information Criterion (AIC). The between-group mean differences estimated from the models at day 30 were adjusted for differences on day 1 and are reported along with their 95% confidence intervals, *t*-values and *p*-values corresponding to the null hypothesis of no between-group differences.

#### 2.7.5. Patient Reported Outcome Measure Analysis

Similar to the Fitbit data, the questionnaire data were also analysed in R using linear regression for the total score and linear mixed regression for questionnaire components. Assumptions of normality and homogeneity of variance of residuals across fitted values were evaluated with QQ plots and fitted-versus-residuals plots. Between-group differences estimated from the models are reported along with their 95% confidence intervals, *t*-values and *p*-values corresponding to the null hypothesis of no between-group differences.

## 3. Results

**Figure 3:**
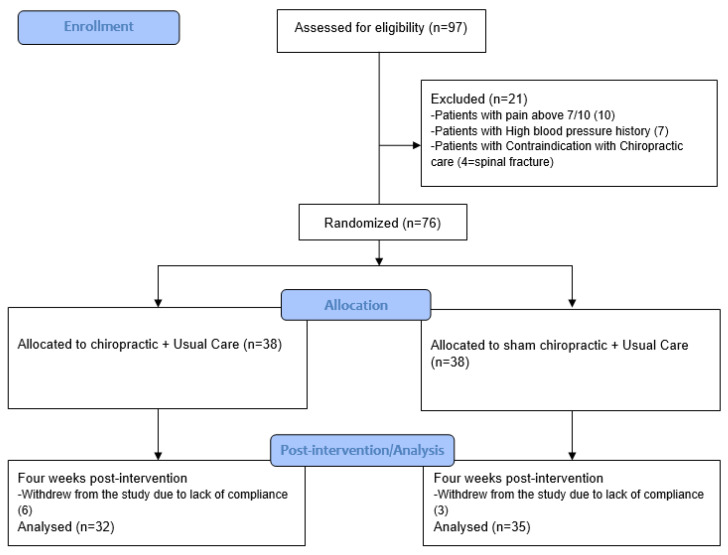
CONSORT flow diagram.

Ninety-seven participants were assessed for eligibility, of which 76 (mean age ± SD: 45 ± 11 years, 33 females) met the eligibility criteria and were enrolled in the study. The participant recruitment flow diagram is given in Figure 3. For participant characteristics, refer to Table 2.

### 3.1. Resting EEG Spectral Power Analysis

**Figure 4:**
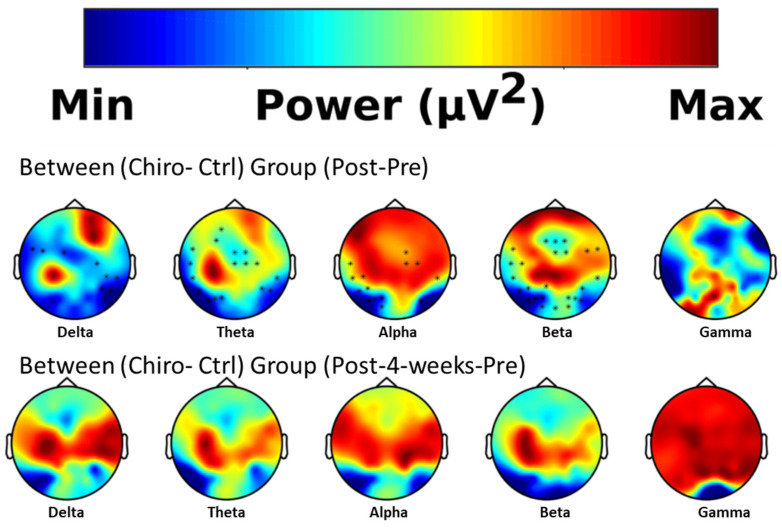
Between-group Spectral analysis. Red indicates increased activity and blue indicates decreased activity. Asterisks represent significant clusters.

The resting EEG spectral power analysis results indicated a significant increase (represented by * in Figure 4) in the Theta, Alpha and Beta band power and a significant decrease in the Delta band in the chiropractic group compared to the control group at post intervention. Although there were noticeable changes in the pre and post 4 weeks between group comparison, none of these changes were statistically significant. These between-group results are shown in Figure 4.

### 3.2. Source Localisation of Resting State EEG

**Figure 5:**
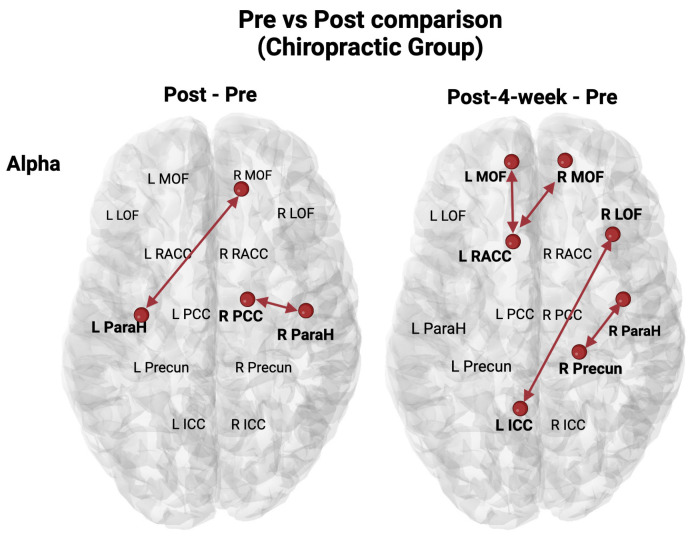
Resting state EEG connectivity analysis of the chiropractic group. A significant increase in connectivity is shown as red. R ICC—Right Isthmus Cingulate Cortex; L ICC—Left Isthmus Cingulate Cortex; R LOF—Right Lateral Orbitofrontal; L LOF—Left Lateral Orbitofrontal; R MOF—Right Medial Orbitofrontal; L MOF—Left Medial Orbitofrontal; R PCC—Right Posterior Cingulate Cortex; L PCC—Left Posterior Cingulate Cortex; R Precun—Right Precuneus; L Precun—Left Precuneus; R ParaH—Right Parahippocampal Cortex; L ParaH—Left Parahippocampal cortex; R RACC—Right Rostral Anterior Cingulate Cortex; L RACC—Left Rostral Anterior Cingulate Cortex.

The resting state EEG analysis revealed an increase in Alpha activity in the chiropractic group after intervention (Pre vs. Post) between (1) the right Parahippocampal cortex and right posterior cingulate cortex and (2) the left Parahippocampal cortex and right medial orbitofrontal. An increase in Alpha activity was also found in the chiropractic group after four weeks of chiropractic care (pre vs. post 4 weeks) between (1) the right Parahippocampal cortex and right Precuneus, (2) the left rostral anterior cingulate cortex and right medial orbitofrontal, (3) the left rostral anterior cingulate cortex and left medial orbitofrontal, and (4) the right lateral orbitofrontal and left isthmus cingulate cortex. These resting state EEG results are shown in Figure 5. No significant changes were found in any other frequency band for the resting state EEG of the chiropractic and the control group. Statistical comparisons for the Alpha band with significant brain regions within the DMN can be found in Table 3.

### 3.3. EEG SEPs Analysis

#### 3.3.1. N30 Peak Amplitude

**Figure 6:**
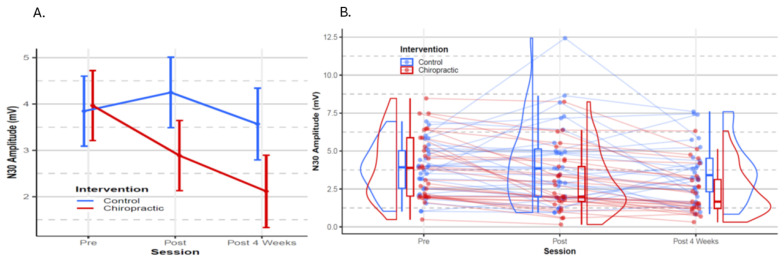
N30 amplitude. (**A**) The N30 SEP peak amplitude changed from baseline to post intervention session and post 4 weeks of intervention. The error bars represent the estimated mean + 95% confidence interval from the statistical model. (**B**) Dots represent N30 amplitude from all participants. Boxplots show the median, 25th and 75th percentiles. The distribution plots show the density distribution estimated by a Gaussian kernel.

The mixed model analysis revealed a significant interaction between the intervention and session (F (2,104) = 7.97, *p* < 0.001) (Table 4). Pairwise comparisons (Table 5) indicated that there was a significant decrease in the N30 amplitude immediately after the chiropractic care (*p* = 0.002). The average N30 amplitude decreased further at post 4 weeks intervention in the chiropractic group (*p* < 0.001). There were no changes observed in the control group (post intervention: *p* = 0.4; at post 4 weeks intervention: *p* = 0.657) (Figure 6).

#### 3.3.2. EEG Source Localisation on SEPs Data

**Figure 7:**
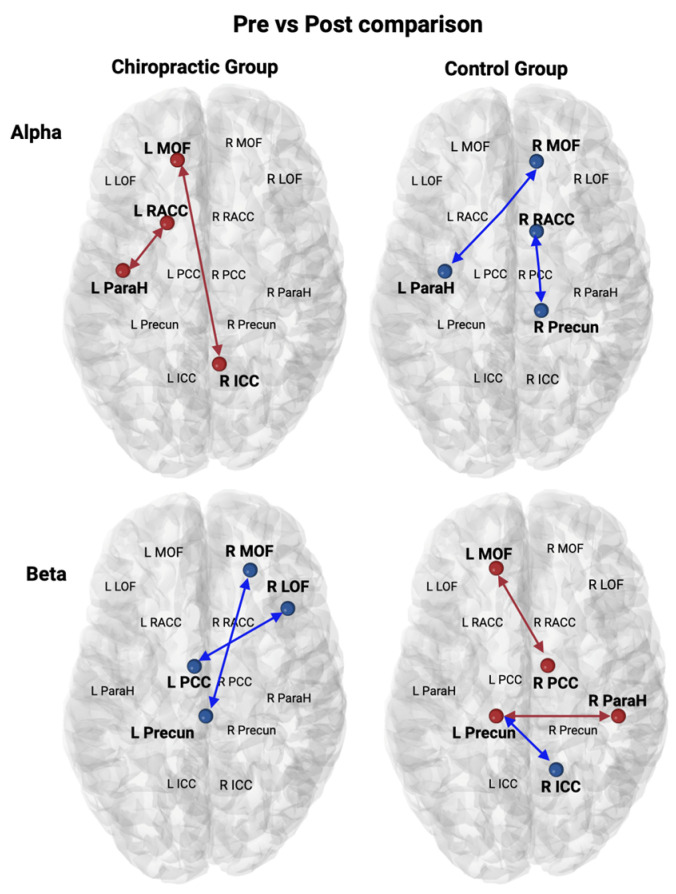
Pre vs. post comparison for control and chiropractic group in SEPs. A significant increase in connectivity is shown as red and a decrease in connectivity is shown as blue. R ICC—Right Isthmus Cingulate Cortex; L ICC—Left Isthmus Cingulate Cortex; R LOF—Right Lateral Orbitofrontal; L LOF— Left Lateral Orbitofrontal; R MOF—Right Medial Orbitofrontal; L MOF—Left Medial Orbitofrontal; R PCC—Right Posterior Cingulate Cortex; L PCC—Left Posterior Cingulate Cortex; R Precun—Right Precuneus; L Precun—Left Precuneus; R ParaH—Right Parahippocampal cortex; L ParaH—Left Parahippocampal cortex; R RACC—Right Rostral Anterior Cingulate Cortex; L RACC—Left Rostral Anterior Cingulate Cortex.

**Figure 8:**
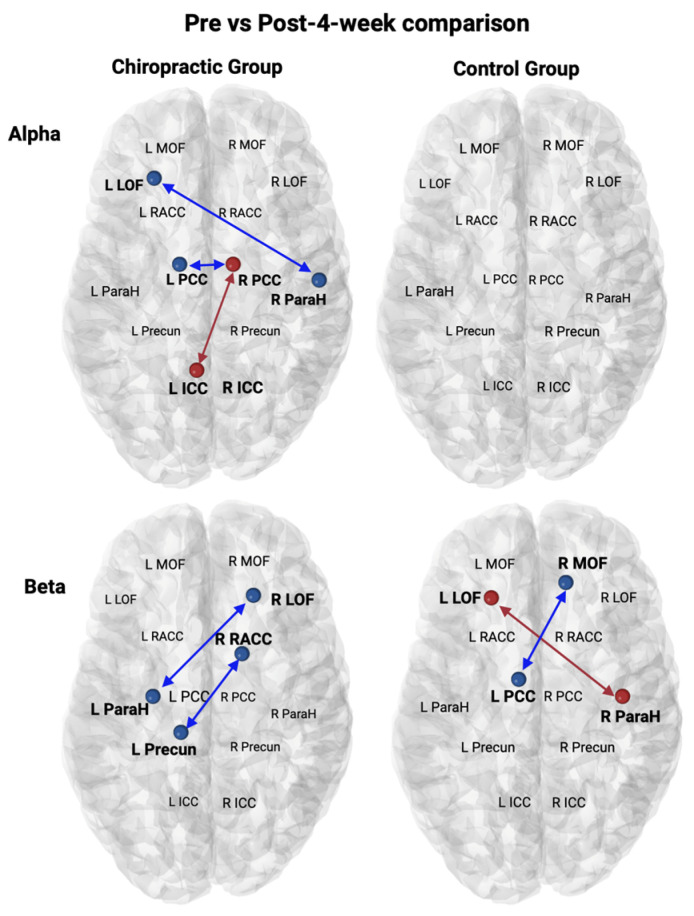
Pre- vs. post 4 weeks comparisons within both groups in SEPs. A significant increase in connectivity is shown as red and a decrease in connectivity is shown as blue. R ICC—Right Isthmus Cingulate Cortex; L ICC—Left Isthmus Cingulate Cortex; R LOF—Right Lateral Orbitofrontal; L LOF—Left Lateral Orbitofrontal; R MOF—Right Medial Orbitofrontal; L MOF—Left Medial Orbitofrontal; R PCC—Right Posterior Cingulate Cortex; L PCC—Left Posterior Cingulate Cortex; R Precun—Right Precuneus; L Precun—Left Precuneus; R ParaH—Right Parahippocampal cortex; L ParaH—Left Parahippocampal Cortex; R RACC—Right Rostral Anterior Cingulate Cortex; L RACC—Left Rostral Anterior Cingulate Cortex.

The chiropractic group showed an increase in Alpha activity and a decrease in Beta activity in SEPs as compared to the control group (Pre vs. Post) (Figure 7). In the chiropractic group, an increase in Alpha activity was found between (1) the right isthmus cingulate cortex and the left medial orbitofrontal and (2) the left Parahippocampal cortex and the left rostral anterior cingulate cortex. A decrease in Beta was found between (1) the left posterior cingulate cortex and the right lateral orbitofrontal cortex and (2) the left precuneus and the right medial orbitofrontal cortex. The pre- vs. post 4 weeks comparison revealed that Alpha activity increased between the left isthmus cingulate cortex and the right posterior cingulate cortex, and decreased between (1) the left lateral orbitofrontal cortex and the right Parahippocampal cortex and (2) the left and right posterior cingulate cortex in the chiropractic group (Figure 8).

Statistical comparisons with both groups for significant frequency bands and brain regions within the DMN can be found in Table 6.

### 3.4. Fitbit Data

**Figure 9:**
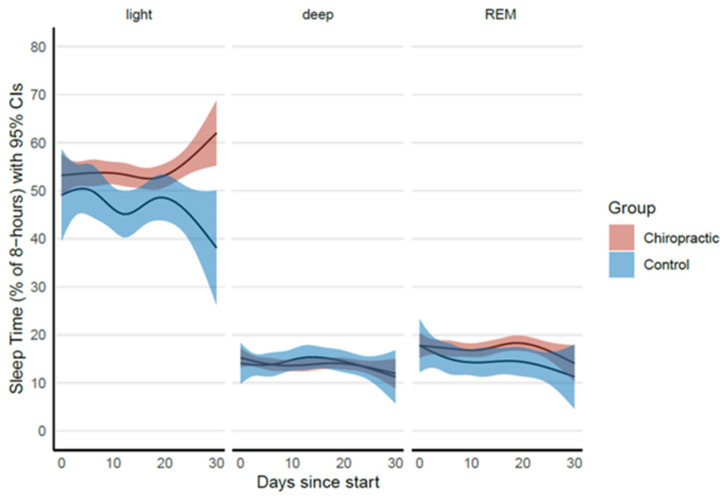
Comparison of sleep stages: light, deep, rapid eye movement (REM)) between groups. Note: Daily sleep time is expressed as a percentage of 8 h.

The Fitbit data were continuously recorded for four weeks. We observed significant differences between the control and chiropractic groups, with the chiropractic group showing significantly increased light sleep stage duration (Table 7). The comparison is shown in Figure 9. There was no significant difference in activity levels between groups.

### 3.5. Patient Reported Outcome Measure

**Figure 10:**
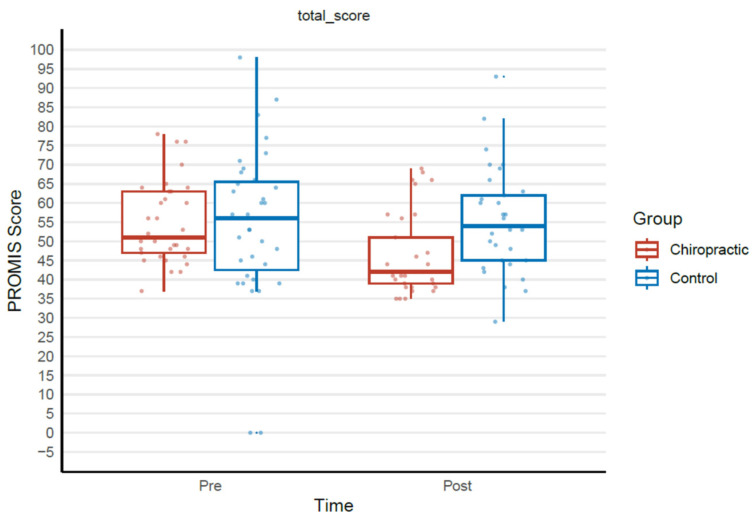
Total quality of life score between groups.

There was an overall improvement in quality of life in the chiropractic care group, as measured by PROMIS-29, compared to the control group. Post 4 weeks, there was a minimal clinically important difference (MCID) [105] between groups of 9.0 in the total score shown in Table 8. The comparison between groups is shown in Figure 10. Chiropractic care had a significant effect on the PROMIS-29 domains of anxiety, depression, fatigue, pain intensity, and pain interference (see details in the Supplementary Materials File S1).

## 4. Discussion

In this comprehensive exploration of the mechanisms of chiropractic care, our study revealed a spectrum of outcomes spanning behavioural, subjective, and neurophysiological domains. Neurophysiological assessments, including EEG spectral power analysis, unveiled a significant increase in the Theta, Alpha, and Beta band power and a significant decrease in Delta straight after the first chiropractic session. Moreover, the N30 SEP peak amplitude decreased post the first chiropractic care session, as previously found in multiple studies [17,21,27,106], and further decreased over the four weeks of chiropractic intervetion. SEPs source connectivity analysis indicated alterations within the DMN activity, particularly an increase in Alpha and a decrease in Beta activity in specific brain regions. Resting state EEG source connectivity analysis further detailed changes in Alpha activity within defined brain regions after the first intervention and after 4 weeks of intervention, also suggesting changes within the DMN. The Fitbit data revealed notable distinctions between the control and chiropractic groups after 4 weeks of the intervention, including an increase in the light sleep stage in the chiropractic group. Subjective assessment using PROMIS-29 highlighted significantly improved overall QOL in the chiropractic group after 4 weeks of care, with significant improvements in the domains of anxiety, depression, fatigue, pain intensity and pain interference. This significant improvement also reached MCID, indicating this improvement in quality of life was clinically meaningful for this group. These results further our understanding of the multifaceted impacts of chiropractic care on various health parameters, as well as improving our understanding about the mechanisms for these changes, as will be discussed below in greater detail.

### 4.1. Resting State EEG Source Localisation

The resting state EEG source localisation connectivity analysis detailed significant changes in Alpha activity within defined brain regions after the first intervention and after 4 weeks of intervention, suggesting changes within the DMN following the chiropractic intervention (Figure 5). No changes were found in the control condition, suggesting these changes within the DMN occurred exclusively following the chiropractic care intervention.

Large-scale brain networks offer a powerful paradigm for understanding and investigating many chronic musculoskeletal, psychiatric, and neurological disorders [107,108,109]. Multiple chronic disorders such as low back pain, anxiety and depression are thought to be caused by aberrant interactions within or between the default mode network, sensory networks, pain networks, the salience network and the fronto-parietal (goal oriented) network [107,108,109]. In the current study, our analysis focused on any possible changes within the DMN, because changes within the DMN had been found in a previous chiropractic study in a chronic stroke population [60]. In this study, in the stroke survivors, there was a significant increase in functional connectivity in the Alpha band within the DMN with increases in functional connectivity between the PCC and ParaH regions [60]. Thus, in the current study, it was imperative to assess whether ‘regular’ chiropractic patients (e.g., CLBP patients with varying degrees of anxiety and depression) also responded to chiropractic care with changes within the DMN. The current study clearly demonstrates that this is the case, with increased Alpha power functional connectivity affecting all the defined regions within the DMN studied. Changes in functional connectivity were found between right ParaH and right PCC, between the left ParaH and the right MOF, between the right ParaH and the right Precun, between right LOF and left ICC, between the left rostral ACC and right MOF and between the left rostral ACC and left MOF.

The DMN involves this collection of brain regions, mentioned above, that are active when a person is not paying attention to an external or internal stimulus or in the absence of goal-oriented thoughts and behaviours [108]. The DMN is active when one engages in self-reflection, mind-wandering, daydreaming, recall of personal experiences, reflection of social interactions and envisaging the future [108]. Chronic problems, such as chronic musculoskeletal pain [109], as well as common chronic disorders such as anxiety and depression [107] have been found to have clear deficits in access, engagement and disengagement of the DMN. Not only have such disorders shown changes within the DMN, but also between the DMN and other networks including various sensory networks, pain networks, the salience network and the fronto-parietal (goal oriented) networks [107,108,109]. The changes within the DMN and between the DMN and other networks provide a neurobiological explanation for why these problems (e.g., pain, anxiety and depression) become embodied, i.e., they become an integral part of the persons sense of self, making treatment difficult [109]. It is thought that when suffering is ongoing long-term the feelings of pain may become part of one’s internal self-story, and, similarly, ongoing worry can develop into anxiety and ongoing sadness can become depression [109]. It is, therefore, fascinating to see that chiropractic care can alter functional connectivity within the DMN in a manner that coincides with improvements in pain, depression, anxiety, fatigue and sleep. It is likely that the functional connectivity changes found within the DMN in the current study between the pre-intervention recording and post 4 weeks of chiropractic care reflects a difference in how the study participants were reflecting on themselves and their everyday experiences over time. The participants appear to be altering their constructed sense of self and how they understand their own mental states over the four weeks of care. At the end of the 4 weeks of chiropractic care, these participants reported significantly less depressive, anxious and pain symptoms. Thus, their self-reflection after 4 weeks of care appears now to be more positive, since they also reported experiencing less pain, less fatigue, less anxiety and less depression and improved sleep.

Alpha rhythms are known to play a key role in scene perception [110] and are also commonly found to be lower in individuals with chronic pain, localised in the insula and particularly in the frontal lobes, when compared with healthy controls [111,112,113,114]. In the current study, there were clear increases in Alpha power connectivity involving the frontal lobes. Thus, the Alpha power frequency changes found in this study likely reflect changes in pain perception in the chiropractic patients. However, some argue that changes in Alpha power may not be exclusively found in pain patients, as it is also found in attention deficit hyperactivity disorder, depression, cognitive function, and with age [114]. It is, therefore, most likely that the change in Alpha activity in our study reflects a combination of changes in processing internal body signals, emotional processing, and spatial awareness after the first adjustment session, resulting in the clinically meaningful improvements found in QOL and sleep over the four weeks of intervention. It is clear, following the current study, that we must also investigate potential changes in other large brain networks, such as the salience network and the fronto-parietal (goal oriented) networks. For a more detailed discussion of the resting state EEG connectivity analysis changes found in the current study, please see Supplementary Materials File S2.

### 4.2. SEPs Measures and SEPs Source Localisation

The N30 SEP peak is associated with various neural generators, including the primary sensory cortex, basal ganglia, thalamus, premotor areas including the prefrontal cortex, and primary motor cortex [115,116]. The PFC, which plays a crucial role in executive functions, is recognised as a significant contributing structure [117]. Any alteration in prefrontal activity after chiropractic care could potentially elucidate or establish connections with the diverse enhancements in neural function previously observed [17,28,29,30,31,33].

The N30 SEP peak amplitude changes that occur with chiropractic care were initially documented in 2007 [17], and repeated in multiple other studies over the next decade [21,27,80,106]. They are thought to reflect an improvement in early sensorimotor integration, that occurs predominantly involving the prefrontal cortex [2,80,118]. In the current study, the N30 SEP peak amplitude was again shown to be decreased both after the first chiropractic session and after the four weeks of chiropractic care. The SEPs source localisation analysis also revealed significant changes in functional connectivity within the DMN following the chiropractic care. This suggests that the altered proprioceptive input from the paraspinal tissues is powerful enough to alter interoception and processing within the DMN.

The chiropractic group showed an increase in Alpha activity and a decrease in Beta activity in the SEP data as compared to the control group (Figure 7). In the chiropractic group, an increase in Alpha activity was found between the right ICC and left MOF, and between the left ParaH and left rostral ACC. A decrease in Beta was found between the left PCC and the right LOF and between the left Precun and the right MOF. After the four weeks of care, there were also changes in Alpha activity with an increased connectivity between the left ICC and right PCC, and decreased connectivity between the left LOF and right ParaH, as well as between left and right PCC in the chiropractic group (Figure 8).

Both the RACC and the ParaH areas are known to be involved in the descending pain inhibitory pathway [109,119,120]. This is particularly interesting, because it is well known that the descending modulatory pain pathway is malfunctional in chronic pain patients [109,121,122,123,124]. In addition, the malfunction of the descending modulatory pain pathway has been implicated in the ‘chronification’ of acute to chronic pain [125]. The current study provides some support for the notion that part of the mechanisms of chiropractic care is that it might improve the descending modulatory pain pathway in CLBP patients, and this may be why it has been found to be clinically effective for both acute and CLBP problems and is a recommended option in relevant clinical guidelines [126,127,128,129,130,131,132,133,134,135,136].

The fact that, yet again, the study participants show clear alterations within the DMN suggest that chiropractic care is altering brain regions that are responsible for self-referential processing and emotional evaluation of internal and external stimuli, and regulation of emotions and memories over the four weeks of chiropractic care. The HVLA adjustments of vertebral subluxations over the four weeks has likely altered the sensory stimuli coming from the spine, and this appears to have changed the way the DMN is assessing the emotional significance of that altered spinal sensory input, thus altering the way the person reflects on their sense of self. Their internal narrative sense of self appears to have been altered over the four weeks of care, with changes in the emotional processing associated with internal and external stimuli which are influencing how they perceive themselves and the world around them, reflected by the reported decrease in fatigue, depression, anxiety, and pain. For a more detailed discussion of the SEPs source localisation connectivity analysis, please see Supplementary Materials File S2.

### 4.3. Resting EEG Spectral Power Analysis

The EEG spectral power analysis results indicated a significant increase in the Theta, Alpha, and Beta band power and a significant decrease in the Delta band in the chiropractic group compared to the control group after the first session of chiropractic care. There were no significant changes after the four weeks of either intervention. Beta power is commonly found to be higher in chronic pain patients [114]. However, the most consistent finding across reviews is an increase in Theta power in chronic pain patients [114]. Alpha power has also been implicated in both acute and chronic pain, and is considered a bio-marker for pain sensitivity, which has been hypothesised to be a key factor in the transition from acute to chronic pain [137,138,139,140]. However, the observed changes in EEG spectral power may not simply reflect changes in pain processing or perception. It is also possible that the observed changes in Beta and Theta power are due to alterations in multimodal sensory integration [141]. In a recent study, Michail and colleagues investigated the influence of limited cognitive resources on audiovisual integration by measuring high-density EEG in healthy participants performing the sound-induced flash illusion [141]. They found changes in both Beta and Theta power to be associated with mismatch signals and subsequent top-down influences involving the ACC, prefrontal cortex, and auditory cortex [141]. Interestingly, a previous chiropractic study has shown that 12 weeks of chiropractic care in older adults significantly improved multimodal sensory integration, also using the sound induced flash illusion [142]. Michail and colleagues suggested that Theta and Beta power band changes might reflect multimodal integration mechanisms that are recruited when the integration of conflicting audiovisual stimuli requires more processing resources [141]. Thus, if multisensory integration engages top-down Theta and Beta oscillations when cognitive resources are scarce, it is possible that the chiropractic adjustments, reducing multimodal mismatch, and thereby improving cognitive resources may have resulted in the reduced Theta and Beta oscillations as seen in our current study.

### 4.4. Fitbit Data

Fitbit data revealed that the light sleep stage significantly increased in duration in the chiropractic group over the four weeks of this study. Improved sleep length is consistent with existing research indicating that chiropractic adjustments help to alleviate tension from within the body, encouraging blood flow, enabling the muscles to relax and making it easier to also mentally relax and fall asleep [143]. As the participants in this study reported improvements in CLBP over the four weeks of the study, the chiropractic adjustments appear to have positively influenced pain processing, another factor known to improve sleep quality. A large USA-based survey previously found that 40% of those who see chiropractors report that they sleep better when under chiropractic care [144]. The current study supports this finding and suggests that chiropractic care does have an impact on sleep quality.

### 4.5. Patient-Reported Outcome Measure

The improvement in overall quality of life measured by PROMIS-29 in the chiropractic group provides evidence that the participants in this study subjectively felt better after four weeks of chiropractic care. This significant improvement also reached MCID, suggesting this improvement in quality of life was clinically meaningful for this group. Numerous previous studies have also reported enhanced quality of life and well-being following chiropractic care, possibly linked to reduced pain and improved mobility [144,145,146,147,148]. However, several studies have found quality of life improvements beyond just improvements in pain or pain related disability [145,148], similar to what has been documented in the current study. In the subsections of the PROMIS-29 tool, there were significant improvements following the four weeks of chiropractic care in anxiety, depression, fatigue, as well as improvements in pain intensity, and pain interference. Major depressive disorder, anxiety disorders, and chronic pain are known to be closely related disorders with both high degrees of comorbidity as well as shared risk factors [149,150]. From a large-scale multimodal meta-analysis, there have been common changes found in intrinsic functional connectivity in people with major depression, anxiety disorders, and chronic pain [149]. These common changes were located mainly within the DMN. The authors argue that such common changes suggest a neural correlate for comorbidity and possibly shared neuro-behavioural ‘chronification’ mechanisms [149]. Our results support this notion, as chiropractic care not only improved symptoms of pain and unpleasantness, but also resulted in improvements in fatigue, anxiety, and depression. Our study adds to a growing body of research pointing to the association between depression and anxiety, and chronic pain, with several mechanisms contributing to a multimodal aetiology for chronic pain in humans [151]. This is important, as pain and pain-related diseases are considered to be the leading causes of disability and the greatest disease burden worldwide [152]. It is estimated that at least 10% of the world’s population is affected by chronic pain (varying from 20% to 25% in some countries and regions). Additionally, each year, approximately one in ten people develop chronic pain [153,154]. The transition from acute to chronic pain results from permanent changes in the nervous system that include peripheral and central sensitisation, central facilitation of nociceptive pathways and dysfunction in descending pain modulatory circuits [155]. Chiropractic care may potentially prevent pain from becoming chronic and the current study provides some insight into the mechanisms of this. For example, the current study changes in functional connectivity between the ACC and other DMN brain regions may reflect changes or increases in the descending pain modulatory circuits, and through these mechanisms provide the relief from pain intensity and unpleasantness. This might be why having ongoing maintenance chiropractic care (i.e., after the initial care period improves symptoms) results in less days of pain and improved QOL on an ongoing basis [156,157,158]. It may also be why those who initiated long-term care for CLBP by seeking chiropractic care compared with initially seeking opioid analgesics experience significantly lower rates of hospitalisations, emergency department visits, advanced diagnostic imaging, specialist visits, lumbosacral surgery, interventional pain medicine techniques, i.e., a reduced escalation of care [159]. Ongoing chiropractic care, even after the initial pain symptoms improve, will likely continue to impact somatosensory networks, the DMN, and, potentially, the Salience Network, thus maintaining positive neuroplastic changes that prevent such escalations of care, worsening of symptoms, and decreases in QOL.

### 4.6. Possible Mechanisms

As discussed above, the results from this study, exploring the impact of chiropractic care on subjective, behavioural, and brain network functional connectivity, help to explain the mechanisms of the wide-ranging clinical improvements observed following chiropractic care. For example, the DMN, which is responsible for self-representational processing has been suggested to become pathologically coupled to pain provoking networks in chronic pain [109]. This provides a neurobiological explanation for why chronic pain becomes embodied and why it lasts beyond the initial injured tissue healing has taken place [109]. If changes within the DMN make pain an integral part of the self, this also makes it very difficult to eliminate. Similarly, when suffering is ongoing (i.e., becomes chronic), not only may the pain become a part of one’s identity, but fear and worry can develop into anxiety problems and sadness can turn into clinical depression [109]. The current findings provide compelling evidence for why chiropractic care, that includes the HVLA adjustments directed towards subluxated segments, can impact not only chronic pain symptoms, but also influence anxiety and depression symptoms.

The contemporary model of the vertebral subluxation suggest that a vertebral subluxation can lead to abnormal multisensory processing and filtering of interoceptive and exteroceptive stimuli that can ultimately lead to poor motor control of the vertebral column as well as other muscles in the body [2]. There is evidence in the literature that proprioceptive input from the deep paraspinal muscles (i.e., those that connect between individual vertebrae) is essential for intervertebral motor control [160]. It is also known that the activity of deep back muscles is different in people with recurrent low back pain, despite the resolution of symptoms [161]. This change in proprioceptive input to the CNS may ultimately be responsible for the development and maintenance of musculoskeletal pain syndromes [162], especially if they become part of the narrative sense of self [109]. This makes sense, as over time, abnormal or reduced proprioceptive input from dysfunctional spinal regions (i.e., vertebral subluxations) would be capable of causing ongoing central maladaptive changes, and the brain’s DMN can embody these ‘perceptions’. Furthermore, with ongoing poor motor control, as the CNS is less accurately aware of what is occurring at the vertebral column level (due to the reduced/abnormal proprioceptive inputs from that paraspinal region); this would likely lead to repeated microtraumas at the subluxated spinal levels as well as other areas of the body that require accurate sensory input from the spine, such as accurate upper and lower limb motor control [2]. Over time, it makes sense that this could explain the development of chronic musculoskeletal pain problems.

This contemporary model also explains how spinal adjustments, i.e., HVLA thrusts delivered to subluxated spinal segments, can improve vertebral column motor control by bombarding the CNS with mechanoreceptive input from the segments that are dysfunctional [75,76,77,78,163]. This is likely a highly salient signal able to activate the brain regions within the Salience Network (which should be explored in future research) to focus attention on the spinal sensory inputs that could lead to changes within the DMN, which is consistent with the current study findings discussed above. It also follows that once the CNS is more accurately aware of the vertebral column, and it changes the body and world schemas, and therefore controls spinal movement better this will also naturally improve whole body perception and functions as well [29,164]. For example, the studies showing improved audio-visual processing or mental rotation task performance following chiropractic care/adjustments support this notion [9,30,142]. Particularly, since there is also evidence to suggest that spinal dysfunction is related to poor audio-visual integration and processing that does not get better over four weeks with no intervention [10]. The results from the current study support this contemporary model and suggest that chiropractic care provided to the study participants has altered the way their brains integrate both interoceptive and exteroceptive sensory information in a multimodal fashion, including the consideration of past memories and future expectations, that have resulted in an altered narrative sense of self (i.e., altered DMN function).

### 4.7. Limitations

Although this research utilised a comprehensive methodology, diverse outcome measures, and a nuanced interpretation of findings, it is important to note certain limitations. For instance, the study relies on EEG data, which, despite its high temporal resolution, low cost and ease of data collection [165], lacks the spatial accuracy of fMRI. Specifically, EEG cannot precisely localize the specific areas within the brain where neural activity originates. The scalp electrodes record the combined electrical activity of numerous neurons, making it difficult to pinpoint exact sources, particularly for signals from deeper brain structures [166]. This limitation leads to challenges in distinguishing overlapping signals from adjacent regions and accurately identifying smaller, specific areas involved in cognitive or emotional processes. For example, the current study cannot reliably locate the Precuneus changes to the anterior or posterior parts. Only an fMRI study could elucidate such nuances. Furthermore, the inverse problem of EEG source localisation is mathematically complex and does not yield unique solutions, introducing potential inaccuracies. EEG is also more sensitive to cortical surface activity and may not fully capture important neural processes in deeper regions like the hippocampus and amygdala, crucial for the affective-emotional and cognitive-evaluative components of chronic pain [167,168]. Additionally, the assumption that specific architectonically defined brain areas have specialised functions is often based on group averages. Individual differences mean that the exact brain regions involved in specific DMN tasks can vary from person to person [169]. It is also important to note that only the DMN was explored. Future studies should also explore possible changes in functional connectivity within the Salience Network and even the Central Executive (Fronto-Parietal) Networks. Therefore, the findings in this study should be interpreted with some caution, and the suggested interpretations will need to be validated in future studies. It is also important to note that there could be some placebo effects impacting the results of the current study, especially after four weeks. There was only a control for placebo interactive effects during the first control intervention. Following this, there was no attempt to control for placebo, as this was not the focus of this study. Furthermore, the absence of long-term follow-up data beyond four weeks is a significant constraint. Acknowledging these limitations is essential, given the potential individual variability in response to chiropractic care due to broad eligibility criteria. Moreover, the study primarily focuses on individuals with CLBP, limiting the generalisability of the findings to broader chiropractic patient populations. Lastly, although rigorous randomisation procedures were used, the study was not registered due to its mechanistic focus. Future research could consider registration. Despite these limitations, this research contributes significantly to the scientific discourse on the mechanisms of chiropractic care, paving the way for further investigations into its holistic impacts on health and well-being.

## 5. Conclusions

This study evaluated the underlying neurophysiological mechanisms of chiropractic care using a multifaceted approach, recording behavioral, subjective, and neurophysiological data. The behavioral data, recorded through Fitbit, revealed a significant improvement in light sleep stage length within the chiropractic care group throughout the 4-week intervention. There was also a significant increase in overall QOL among chiropractic care participants, with clinically meaningful improvements in anxiety, depression, fatigue, as well as decreases in pain intensity, and pain interference. These subjective and behavioral improvements were recorded alongside neurophysiological findings obtained through EEG spectral and source-level analysis. Chiropractic adjustments most likely act as a salient signal that is able to activate the Salience Network, so that the CNS focuses attention on the vertebral column sensory inputs. This needs to be explored in future analyses. All of the results in this study suggest there was an increased attention towards the spinal proprioceptive input from the adjustments that have altered the way the participants brains integrated both interoceptive and exteroceptive sensory information in a multimodal fashion, including the consideration of past memories and future expectations, that have resulted in an altered narrative sense of self, i.e., altered DMN function that has resulted in improved symptoms and better health. It suggests that once the CNS is more accurately aware of the vertebral column, it has resulted in updated body and world schemas, thus the CNS will be able to control vertebral column movement better, reducing microtraumas and improving function and symptomatology. Over the four weeks of care, this altered DMN function and improved body schema and external world schema appear to have enabled improved overall whole body perception, emotional control and functions as well, reflected in less pain intensity and less unpleasantness, less anxiety, less depression, increased energy, and more light sleep and light activity. These results provide support for the contemporary model of the vertebral subluxation and help to explain how chiropractic HVLA adjustments impact the way the brain integrates both interoceptive and exteroceptive sensory information in a multimodal fashion, including the consideration of past memories and future expectations. The current study’s findings within the DMN suggest that chiropractic care alters a person’s narrative sense of self, that can enable that person to think, feel and function better.

## Figures and Tables

**Table 1:** Default Mode Network brain regions and their abbreviations.

Brain Regions	Abbreviations
Right Medial Orbitofrontal	R MOF
Left Medial Orbitofrontal	L MOF
Right Lateral Orbitofrontal	R LOF
Left Lateral Orbitofrontal	L LOF
Right Parahippocampal	R ParaH
Left Parahippocampal	L ParaH
Right Isthmus Cingulate Cortex	R ICC
Left Isthmus Cingulate Cortex	L ICC
Right Precuneus	R Precun
Left Precuneus	L Precun
Right Posterior Cingulate Cortex	R PCC
Left Posterior Cingulate Cortex	L PCC
Right Rostral Anterior Cingulate Cortex	R RACC
Left Rostral Anterior Cingulate Cortex	L RACC

**Table 2:** Characteristics of participants who completed the full study.

Group	Chiro	Control
Age (mean ± SD, years)	46 ± 11	42 ± 9
Gender (F:M)	19:13	14:21

Legend: Female (F), male (M), standard deviation (SD).

**Table 3:** Statistical information within DMN for resting state EEG. R ICC—Right Isthmus Cingulate Cortex, L ICC—Left Isthmus Cingulate Cortex; R LOF—Right Lateral Orbitofrontal; L LOF—Left Lateral Orbitofrontal; R MOF—Right Medial Orbitofrontal; L MOF—Left Medial Orbitofrontal; R PCC—Right Posterior Cingulate Cortex; L PCC—Left Posterior Cingulate Cortex; R Precun—Right Precuneus; L Precun—Left Precuneus; R ParaH—Right Parahippocampal cortex; L ParaH—Left Parahippocampal Cortex; R RACC—Right Rostral Anterior Cingulate Cortex; L RACC—Left Rostral Anterior Cingulate Cortex; PLI—Phase lag index.

Group	Sessions	Frequency Band	Regions	*t*-Value	*p*-Value	Mean Difference PLI
Chiro	Post–Pre	Alpha	R PCC–R ParaH	0.43	0.03	0.004
		Alpha	R MOF–L ParaH	1.46	0.02	0.01
	Post 4 weeks–Pre	Alpha	R ParaH–R Precun	0.26	0.04	0.002
		Alpha	L RACC–R MOF	0.64	0.02	0.006
		Alpha	L RACC–L MOF	0.42	0.03	0.003
		Alpha	R LOF–L ICC	0.5	0.01	0.002

**Table 4:** Model results.

	F	Df	Df.res	Pr (>F)
Intervention	3.26	1	55.94	0.077
Session	11.08	2	104.93	<0.001
Intervention: Session	7.97	2	104.93	<0.001

**Table 5:** Differences between groups based on estimated N30 amplitude. SE: Standard error, CL: Confidence limit.

Group	Contrast	Estimate	SE	Lower CL	Upper CL	t-Ratio	*p*-Value
Chiro	Post–Pre	−1.08	0.31	−1.81	−0.35	−3.52	0.002
Chiro	Post 4 weeks–Pre	−1.85	0.32	−2.62	−1.08	−5.72	<0.001
Control	Post–Pre	0.40	0.31	−0.34	1.14	1.30	0.40
Control	Post 4 weeks–Pre	−0.28	0.32	−1.04	0.48	−0.88	0.66

**Table 6:** Statistical information within DMN for SEPs. R ICC—Right Isthmus Cingulate Cortex; L ICC—Left Isthmus Cingulate Cortex; R LOF: Right Lateral Orbitofrontal; L LOF: Left Lateral Orbitofrontal; R MOF: Right Medial Orbitofrontal; L MOF: Left Medial Orbitofrontal; R PCC: Right Posterior Cingulate Cortex; L PCC: Left Posterior Cingulate Cortex; R Precun: Right Precuneus, L Precun; Left Precuneus: R ParaH; Right Parahippocampal Cortex; L ParaH: Left Parahippocampal Cortex; R RACC: Right Rostral Anterior Cingulate Cortex; L RACC: Left Rostral Anterior Cingulate Cortex; PLI: Phase lag index.

Group	Sessions	Frequency Band	Brain Regions	*t*-Value	*p*-Value	Mean PLI Difference
Chiro	Post–Pre	Alpha	L ParaH–L RACC	1.04	0.043	0.03
		Alpha	R ICC–L MOF	0.29	0.03	0.006
	Post 4 weeks–Pre	Alpha	L PCC–RPCC	−0.62	0.017	−0.027
		Alpha	RPCC–L ICC	0.29	0.009	0.007
		Alpha	R ParaH–L LOF	−0.28	0.03	−0.0069
	Post–Pre	Beta	RMOF—L Precun	−2.43	0.02	−0.061
		Beta	R LOF–L PCC	−2.21	0.035	−0.074
	Post 4 weeks–Pre	Beta	L Precun–R RACC	−0.4	0.04	−0.01
		Beta	R LOF–L ParaH	0.08	0.009	−0.0022
Control	Post–Pre	Alpha	R Precun–R RACC	−0.36	0.011	−0.007
		Alpha	L ParaH RMOF	−0.76	0.04	−0.018
	Post–Pre	Beta	L MOF–RPCC	1.2	0.02	0.04
		Beta	L Precun–RICC	−0.36	0.007	−0.011
		Beta	L Precun–R ParaH	0.81	0.004	0.02
	Post 4 weeks–Pre	Beta	L LOF–R ParaH	1.66	0.038	0.05
		Beta	L PCC–R MOF	−3.74	0.031	−0.1

**Table 7:** Between-group differences in sleep stage duration expressed as a percentage of 8 h.

Contrast	Difference ± SE (95% CI)	*t* (df), *p*-Value
Light [Chiro-Control]	20 ± 8 (5, 35)	*t* (2438) = 2.54, 0.01
Deep [Chiro-Control]	0 ± 3 (−7, 6)	*t* (2438) = −0.13, 0.90
REM [Chiro-Control]	3 ± 4 (−6, 11)	*t* (2438) = 0.65, 0.52

**Table 8:** Between-group differences in total quality of life score.

Contrast	Difference ± SE (95% CI)	*t* (df), *p*-Value
PROMIS Score [Chiro-Control]	−9 ± 3 (−15, −4)	*t* (63) = −3.31, 0.002

## Data Availability

Reasonable request for data can be requested from corresponding author but we will need to seek ethics committee approval prior to sharing any data.

## Supplementary Materials

The following supporting information can be downloaded at: https://www.mdpi.com/article/10.3390/brainsci14111124/s1. File S1: Results of PROMIS-29 domains of anxiety, depression, fatigue, pain intensity, and pain interference has been attached and File S2: Detailed discussion about EEG and SEPs source localisation changes.

## References

[1] Haavik H., Murphy B. (2012). The role of spinal manipulation in addressing disordered sensorimotor integration and altered motor control. J. Electromyogr. Kinesiol..

[2] Haavik H., Kumari N., Holt K., Niazi I.K., Amjad I., Pujari A.N., Türker K.S., Murphy B. (2021). The contemporary model of vertebral column joint dysfunction and impact of high-velocity, low-amplitude controlled vertebral thrusts on neuromuscular function. Eur. J. Appl. Physiol..

[3] Rosner A.L. (2016). Chiropractic identity: A neurological, professional, and political assessment. J. Chiropr. Humanit..

[4] World Health Organization (2005). WHO Guidelines on Basic Training and Safety in Chiropractic.

[5] Holt K., Russell D., Cooperstein R., Young M., Sherson M., Haavik H. (2018). Interexaminer reliability of a multidimensional battery of tests used to assess for vertebral subluxations. Chiropr. J. Aust..

[6] Triano J.J., Budgell B., Bagnulo A., Roffey B., Bergmann T., Cooperstein R., Gleberzon B., Good C., Perron J., Tepe R. (2013). Review of methods used by chiropractors to determine the site for applying manipulation. Chiropr. Man. Ther..

[7] Taylor H.H., Holt K., Murphy B. (2010). Exploring the Neuromodulatory Effects of the Vertebral Subluxation and Chiropractic Care. Chiropr. J. Aust..

[8] Andrew D., Yielder P., Haavik H., Murphy B. (2018). The effects of subclinical neck pain on sensorimotor integration following a complex motor pursuit task. Exp. Brain Res..

[9] Baarbé J.K., Holmes M.W., Murphy H.E., Haavik H., Murphy B.A. (2016). Influence of subclinical neck pain on the ability to perform a mental rotation task: A 4-week longitudinal study with a healthy control group comparison. J. Manip. Physiol. Ther..

[10] Farid B., Yielder P., Holmes M., Haavik H., Murphy B.A. (2018). Association of subclinical neck pain with altered multisensory integration at baseline and 4-week follow-up relative to asymptomatic controls. J. Manip. Physiol. Ther..

[11] The Rubicon Group (2017). Definition and Position Statement on the Chiropractic Subluxation.

[12] Pickar J.G. (2002). Neurophysiological effects of spinal manipulation. Spine J..

[13] Boal R.W., Gillette R.G. (2004). Central neuronal plasticity, low back pain and spinal manipulative therapy. J. Manip. Physiol. Ther..

[14] Gyer G., Michael J., Inklebarger J., Tedla J.S. (2019). Spinal manipulation therapy: Is it all about the brain? A current review of the neurophysiological effects of manipulation. J. Integr. Med..

[15] Christiansen T.L., Niazi I.K., Holt K., Nedergaard R.W., Duehr J., Allen K., Marshall P., Türker K.S., Hartvigsen J., Haavik H. (2018). The effects of a single session of spinal manipulation on strength and cortical drive in athletes. Eur. J. Appl. Physiol..

[16] Daligadu J., Haavik H., Yielder P.C., Baarbe J., Murphy B. (2013). Alterations in cortical and cerebellar motor processing in subclinical neck pain patients following spinal manipulation. J. Manip. Physiol. Ther..

[17] Haavik-Taylor H., Murphy B. (2007). Cervical spine manipulation alters sensorimotor integration: A somatosensory evoked potential study. Clin. Neurophysiol..

[18] Niazi I.K., Türker K.S., Flavel S., Kinget M., Duehr J., Haavik H. (2015). Changes in H-reflex and V-waves following spinal manipulation. Exp. Brain Res..

[19] Haavik H., Niazi I.K., Jochumsen M., Uginčius P., Sebik O., Yılmaz G., Navid M.S., Özyurt M.G., Türker K.S. (2018). Chiropractic spinal manipulation alters TMS induced I-wave excitability and shortens the cortical silent period. J. Electromyogr. Kinesiol..

[20] Holt K., Niazi I.K., Nedergaard R.W., Duehr J., Amjad I., Shafique M., Anwar M.N., Ndetan H., Turker K.S., Haavik H. (2019). The effects of a single session of chiropractic care on strength, cortical drive, and spinal excitability in stroke patients. Sci. Rep..

[21] Haavik H., Niazi I.K., Holt K., Murphy B. (2017). Effects of 12 Weeks of Chiropractic Care on Central Integration of Dual Somatosensory Input in Chronic Pain Patients: A Preliminary Study. J. Manip. Physiol. Ther..

[22] Niazi I.K., Navid M.S., Merkle C., Amjad I., Kumari N., Trager R.J., Holt K., Haavik H. (2024). A randomized controlled trial comparing different sites of high-velocity low amplitude thrust on sensorimotor integration parameters. Sci. Rep..

[23] Robinault L., Holobar A., Crémoux S., Rashid U., Niazi I.K., Holt K., Lauber J., Haavik H. (2021). The Effects of Spinal Manipulation on Motor Unit Behavior. Brain Sci..

[24] Asan A.S., McIntosh J.R., Carmel J.B. (2022). Targeting sensory and motor integration for recovery of movement after CNS injury. Front. Neurosci..

[25] Uysal S.A., Düger T. (2020). Motor control and sensory-motor integration of human movement. Comparative Kinesiology of the Human Body.

[26] Holt K., Niazi I.K., Amjad I., Kumari N., Rashid U., Duehr J., Navid M.S., Shafique M., Haavik H. (2021). The Effects of 4 Weeks of Chiropractic Spinal Adjustments on Motor Function in People with Stroke: A Randomized Controlled Trial. Brain Sci..

[27] Haavik Taylor H., Murphy B. (2010). Altered Central Integration of Dual Somatosensory Input Following Cervical Spine Manipulation. J. Manip. Physiol. Ther..

[28] Taylor H.H., Murphy B. (2008). Altered Sensorimotor Integration With Cervical Spine Manipulation. J. Manip. Physiol. Ther..

[29] Haavik H., Murphy B. (2011). Subclinical neck pain and the effects of cervical manipulation on elbow joint position sense. J. Manip. Physiol. Ther..

[30] Kelly D.D., Murphy B.A., Backhouse D.P. (2000). Use of a mental rotation reaction-time paradigm to measure the effects of upper cervical adjustments on cortical processing: A pilot study. J. Manip. Physiol. Ther..

[31] Herzog W., Scheele D., Conway P.J. (1999). Electromyographic responses of back and limb muscles associated with spinal manipulative therapy. Spine.

[32] Cleland J., Selleck B., Stowell T., Browne L., Alberini S., St. Cyr H., Caron T. (2004). Short-term effects of thoracic manipulation on lower trapezius muscle strength. J. Man. Manip. Ther..

[33] Hillermann B., Gomes A.N., Korporaal C., Jackson D. (2006). A pilot study comparing the effects of spinal manipulative therapy with those of extra-spinal manipulative therapy on quadriceps muscle strength. J. Manip. Physiol. Ther..

[34] Kingett M., Holt K., Niazi I.K., Nedergaard R.W., Lee M., Haavik H. (2019). Increased Voluntary Activation of the Elbow Flexors Following a Single Session of Spinal Manipulation in a Subclinical Neck Pain Population. Brain Sci..

[35] Niazi I.K., Kamavuako E.N., Holt K., Janjua T.A.M., Kumari N., Amjad I., Haavik H. (2020). The Effect of Spinal Manipulation on the Electrophysiological and Metabolic Properties of the Tibialis Anterior Muscle. Healthcare.

[36] Kummer K.K., Mitrić M., Kalpachidou T., Kress M. (2020). The medial prefrontal cortex as a central hub for mental comorbidities associated with chronic pain. Int. J. Mol. Sci..

[37] Chaminade T., Meltzoff A.N., Decety J. (2005). An fMRI study of imitation: Action representation and body schema. Neuropsychologia.

[38] Faw B. (2003). Pre-frontal executive committee for perception, working memory, attention, long-term memory, motor control, and thinking: A tutorial review. Conscious. Cogn..

[39] Manzoni D. (2005). The cerebellum may implement the appropriate coupling of sensory inputs and motor responses: Evidence from vestibular physiology. Cerebellum.

[40] Ochsner K.N., Ray R.D., Cooper J.C., Robertson E.R., Chopra S., Gabrieli J.D., Gross J.J. (2004). For better or for worse: Neural systems supporting the cognitive down-and up-regulation of negative emotion. Neuroimage.

[41] Phillips J.R., Hewedi D.H., Eissa A.M., Moustafa A.A. (2015). The cerebellum and psychiatric disorders. Front. Public Health.

[42] Eden A.S., Schreiber J., Anwander A., Keuper K., Laeger I., Zwanzger P., Zwitserlood P., Kugel H., Dobel C. (2015). Emotion regulation and trait anxiety are predicted by the microstructure of fibers between amygdala and prefrontal cortex. J. Neurosci..

[43] Buijs R.M., Van Eden C.G. (2000). The integration of stress by the hypothalamus, amygdala and prefrontal cortex: Balance between the autonomic nervous system and the neuroendocrine system. Prog. Brain Res..

[44] Liberzon I., King A.P., Britton J.C., Phan K.L., Abelson J.L., Taylor S.F. (2007). Paralimbic and medial prefrontal cortical involvement in neuroendocrine responses to traumatic stimuli. Am. J. Psychiatry.

[45] Hänsel A., Von Känel R. (2008). The ventro-medial prefrontal cortex: A major link between the autonomic nervous system, regulation of emotion, and stress reactivity?. BioPsychoSocial Med..

[46] Sklerov M., Dayan E., Browner N. (2019). Functional neuroimaging of the central autonomic network: Recent developments and clinical implications. Clin. Auton. Res..

[47] Thayer J.F., Sternberg E.M. (2010). Neural aspects of immunomodulation: Focus on the vagus nerve. Brain Behav. Immun..

[48] Rizzi A., Saccia M., Benagiano V. (2020). Is the Cerebellum Involved in the Nervous Control of the Immune System Function?. Endocr. Metab. Immune Disord.-Drug Targets.

[49] Norman K.A., O’Reilly R.C. (2003). Modeling hippocampal and neocortical contributions to recognition memory: A complementary-learning-systems approach. Psychol. Rev..

[50] Šverko Z., Vrankić M., Vlahinić S., Rogelj P. (2022). Complex Pearson correlation coefficient for EEG connectivity analysis. Sensors.

[51] Szczepanski S.M., Pinsk M.A., Douglas M.M., Kastner S., Saalmann Y.B. (2013). Functional and structural architecture of the human dorsal frontoparietal attention network. Proc. Natl. Acad. Sci. USA.

[52] Gay C.W., Robinson M.E., George S.Z., Perlstein W.M., Bishop M.D. (2014). Immediate changes after manual therapy in resting-state functional connectivity as measured by functional magnetic resonance imaging in participants with induced low back pain. J. Manip. Physiol. Ther..

[53] Mateos D.M., Krumm G., Arán Filippetti V., Gutierrez M. (2022). Power spectrum and connectivity analysis in EEG recording during attention and creativity performance in children. NeuroSci.

[54] Ueda R., Takeichi H., Kaga Y., Oguri M., Saito Y., Nakagawa E., Maegaki Y., Inagaki M. (2020). Atypical gamma functional connectivity pattern during light sleep in children with attention deficit hyperactivity disorder. Brain Dev..

[55] Duan W., Chen X., Wang Y.-J., Zhao W., Yuan H., Lei X. (2021). Reproducibility of power spectrum, functional connectivity and network construction in resting-state EEG. J. Neurosci. Methods.

[56] Hassan M., Wendling F. (2018). Electroencephalography source connectivity: Aiming for high resolution of brain networks in time and space. IEEE Signal Process. Mag..

[57] Rutkove S.B. (2007). Introduction to volume conduction. The Clinical Neurophysiology Primer.

[58] Briels C.T., Schoonhoven D.N., Stam C.J., de Waal H., Scheltens P., Gouw A.A. (2020). Reproducibility of EEG functional connectivity in Alzheimer’s disease. Alzheimer’s Res. Ther..

[59] Navid M.S., Lelic D., Niazi I.K., Holt K., Mark E.B., Drewes A.M., Haavik H. (2019). The effects of chiropractic spinal manipulation on central processing of tonic pain—A pilot study using standardized low-resolution brain electromagnetic tomography (sLORETA). Sci. Rep..

[60] Steven Waterstone T., Niazi I.K., Navid M.S., Amjad I., Shafique M., Holt K., Haavik H., Samani A. (2020). Functional Connectivity Analysis on Resting-State Electroencephalography Signals Following Chiropractic Spinal Manipulation in Stroke Patients. Brain Sci..

[61] Dionne C.E., Dunn K.M., Croft P.R., Nachemson A.L., Buchbinder R., Walker B.F., Wyatt M., Cassidy J.D., Rossignol M., Leboeuf-Yde C. (2008). A consensus approach toward the standardization of back pain definitions for use in prevalence studies. Spine.

[62] Bernell S., Howard S.W. (2016). Use your words carefully: What is a chronic disease?. Front. Public Health.

[63] Maher C., Underwood M., Buchbinder R. (2017). Non-specific low back pain. Lancet.

[64] Goertz C.M., Long C.R., Vining R.D., Pohlman K.A., Walter J., Coulter I. (2018). Effect of usual medical care plus chiropractic care vs usual medical care alone on pain and disability among US service members with low back pain: A comparative effectiveness clinical trial. JAMA Netw. Open.

[65] Navid M.S., Niazi I.K., Lelic D., Nedergaard R.B., Holt K., Amjad I., Drewes A.M., Haavik H. (2020). Investigating the Effects of Chiropractic Spinal Manipulation on EEG in Stroke Patients. Brain Sci..

[66] Haavik H., Niazi I.K., Amjad I., Kumari N., Rashid U., Duehr J., Navid M.S., Trager R.J., Shafique M., Holt K. (2022). The Effects of Four Weeks of Chiropractic Spinal Adjustments on Blood Biomarkers in Adults with Chronic Stroke: Secondary Outcomes of a Randomized Controlled Trial. J. Clin. Med..

[67] Cooperstein R., Gleberzon B. (2004). Technique Systems in Chiropractic.

[68] Reed W.R., Cao D.-Y., Long C.R., Kawchuk G.N., Pickar J.G. (2013). Relationship between biomechanical characteristics of spinal manipulation and neural responses in an animal model: Effect of linear control of thrust displacement versus force, thrust amplitude, thrust duration, and thrust rate. Evid.-Based Complement. Altern. Med..

[69] Reed W.R., Long C.R., Pickar J.G. (2013). Effects of unilateral facet fixation and facetectomy on muscle spindle responsiveness during simulated spinal manipulation in an animal model. J. Manip. Physiol. Ther..

[70] Reed W.R., Long C.R., Kawchuk G.N., Pickar J.G. (2014). Neural responses to the mechanical parameters of a high-velocity, low-amplitude spinal manipulation: Effect of preload parameters. J. Manip. Physiol. Ther..

[71] Reed W.R., Pickar J.G., Sozio R.S., Long C.R. (2014). Effect of spinal manipulation thrust magnitude on trunk mechanical activation thresholds of lateral thalamic neurons. J. Manip. Physiol. Ther..

[72] Reed W.R., Pickar J.G. (2015). Paraspinal Muscle Spindle Response to Intervertebral Fixation and Segmental Thrust Level During Spinal Manipulation in an Animal Model. Spine (1976).

[73] Reed W.R., Cranston J.T., Onifer S.M., Little J.W., Sozio R.S. (2017). Decreased spontaneous activity and altered evoked nociceptive response of rat thalamic submedius neurons to lumbar vertebra thrust. Exp. Brain Res..

[74] Reed W.R., Pickar J.G., Sozio R.S., Liebschner M.A., Little J.W., Gudavalli M.R. (2017). Characteristics of Paraspinal muscle spindle response to mechanically assisted spinal manipulation: A preliminary report. J. Manip. Physiol. Ther..

[75] Pickar J.G., Wheeler J.D. (2001). Response of muscle proprioceptors to spinal manipulative-like loads in the anesthetized cat. J. Manip. Physiol. Ther..

[76] Sung P.S., Kang Y.M., Pickar J.G. (2005). Effect of spinal manipulation duration on low threshold mechanoreceptors in lumbar paraspinal muscles: A preliminary report. Spine (1976).

[77] Pickar J.G., Kang Y.M. (2006). Paraspinal muscle spindle responses to the duration of a spinal manipulation under force control. J. Manip. Physiol. Ther..

[78] Cao D., Reed W., Long C., Kawchuk G., Pickar J. (2013). Effects of thrust amplitude and duration of high-velocity, low-amplitude spinal manipulation on lumbar muscle spindle responses to vertebral position and movement. J. Manip. Physiol. Ther..

[79] Homan R.W. (1988). The 10-20 electrode system and cerebral location. Am. J. EEG Technol..

[80] Lelic D., Niazi I.K., Holt K., Jochumsen M., Dremstrup K., Yielder P., Murphy B., Drewes A.M., Haavik H. (2016). Manipulation of dysfunctional spinal joints affects sensorimotor integration in the prefrontal cortex: A brain source localization study. Neural Plast..

[81] Navid M.S., Niazi I.K., Lelic D., Drewes A.M., Haavik H. (2019). The Effects of Filter’s Class, Cutoff Frequencies, and Independent Component Analysis on the Amplitude of Somatosensory Evoked Potentials Recorded from Healthy Volunteers. Sensors.

[82] Nazari G., MacDermid J.C., Sinden K.E., Richardson J., Tang A. (2019). Reliability of Zephyr Bioharness and Fitbit Charge measures of heart rate and activity at rest, during the modified Canadian aerobic fitness test, and recovery. J. Strength Cond. Res..

[83] Fitabase. Data Resolutions. https://www.fitabase.com/resources/knowledge-base/learn-about-fitbit-data/data-resolutions/.

[84] Hays R.D., Spritzer K.L., Schalet B.D., Cella D. (2018). PROMIS((R))-29 v2.0 profile physical and mental health summary scores. Qual. Life Res..

[85] Edit V., Eva S., Maria K., Istvan R., Agnes C., Zsolt N., Eva P., Laszlo H., Peter T.I., Emese K. (2013). Psychosocial, educational, and somatic factors in chronic nonspecific low back pain. Rheumatol. Int..

[86] Singhal K., Muliyala K.P., Pakhare A.P., Behera P., Santoshi J.A. (2021). Do patients of chronic low back pain have psychological comorbidities?. Avicenna J. Med..

[87] Delorme A., Makeig S. (2004). EEGLAB: An open source toolbox for analysis of single-trial EEG dynamics including independent component analysis. J. Neurosci. Methods.

[88] Lopez-Calderon J., Luck S.J. (2014). ERPLAB: An open-source toolbox for the analysis of event-related potentials. Front. Hum. Neurosci..

[89] Bigdely-Shamlo N., Mullen T., Kothe C., Su K.-M., Robbins K.A. (2015). The PREP pipeline: Standardized preprocessing for large-scale EEG analysis. Front. Neuroinform..

[90] Tadel F., Baillet S., Mosher J.C., Pantazis D., Leahy R.M. (2011). Brainstorm: A user-friendly application for MEG/EEG analysis. Comput. Intell. Neurosci..

[91] Hämäläinen M.S., Ilmoniemi R.J. (1994). Interpreting magnetic fields of the brain: Minimum norm estimates. Med. Biol. Eng. Comput..

[92] Friston K. (2011). Dynamic causal modeling and Granger causality Comments on: The identification of interacting networks in the brain using fMRI: Model selection, causality and deconvolution. Neuroimage.

[93] Sadleir R., Argibay A. (2007). Modeling skull electrical properties. Ann. Biomed. Eng..

[94] Schoffelen J.M., Gross J. (2009). Source connectivity analysis with MEG and EEG. Hum. Brain Mapp..

[95] Grech R., Cassar T., Muscat J., Camilleri K.P., Fabri S.G., Zervakis M., Xanthopoulos P., Sakkalis V., Vanrumste B. (2008). Review on solving the inverse problem in EEG source analysis. J. Neuroeng. Rehabil..

[96] Brunovsky M., Krajca V., Diblikova F., Bartos A., Zavesicka L., Matousek M. (2008). Standardized low-resolution brain electromagnetic tomography (sLORETA) in the prediction of response to cholinesterase inhibitors in patients with Alzheimer’s disease. Ann. Gen. Psychiatry.

[97] Kabbara A., El Falou W., Khalil M., Wendling F., Hassan M. (2017). The dynamic functional core network of the human brain at rest. Sci. Rep..

[98] Newson J.J., Thiagarajan T.C. (2019). EEG frequency bands in psychiatric disorders: A review of resting state studies. Front. Hum. Neurosci..

[99] U.S. Center for Disease Control and Prevention. https://www.cdc.gov/sleep/about/?CDC_AAref_Val=https://www.cdc.gov/sleep/about_sleep/how_much_sleep.html.

[100] Waller L., Brovkin A., Dorfschmidt L., Bzdok D., Walter H., Kruschwitz J.D. (2018). GraphVar 2.0: A user-friendly toolbox for machine learning on functional connectivity measures. J. Neurosci. Methods.

[101] Goutte C., Toft P., Rostrup E., Nielsen F.Å., Hansen L.K. (1999). On clustering fMRI time series. NeuroImage.

[102] Bates D., Maechler M., Bolker B., Walker S., Christensen R., Singmann H., Dai B., Scheipl F., Grothendieck G., Green P. (2020). lme4: Linear Mixed-Effects Models Using Eigen and S4.

[103] R Development Core Team (2010). R: A Language and Environment for Statistical Computing.

[104] Russell L. (2020). Emmeans: Estimated Marginal Means, aka Least-Squares Means.

[105] Khutok K., Janwantanakul P., Jensen M.P., Kanlayanaphotporn R. (2021). Responsiveness of the PROMIS-29 Scales in Individuals With Chronic Low Back Pain. Spine (1976).

[106] Haavik Taylor H., Murphy B. (2010). The effects of spinal manipulation on central integration of dual somatosensory input observed following motor training: A crossover study. J. Manip. Physiol. Ther..

[107] Menon V. (2011). Large-scale brain networks and psychopathology: A unifying triple network model. Trends Cogn. Sci..

[108] Menon V. (2023). 20 years of the default mode network: A review and synthesis. Neuron.

[109] De Ridder D., Vanneste S., Smith M., Adhia D. (2022). Pain and the triple network model. Front. Neurol..

[110] Stecher R., Kaiser D. (2024). Representations of imaginary scenes and their properties in cortical alpha activity. Sci. Rep..

[111] Stern J., Jeanmonod D., Sarnthein J. (2006). Persistent EEG overactivation in the cortical pain matrix of neurogenic pain patients. Neuroimage.

[112] Jensen M., Sherlin L., Gertz K., Braden A., Kupper A., Gianas A., Howe J., Hakimian S. (2013). Brain EEG activity correlates of chronic pain in persons with spinal cord injury: Clinical implications. Spinal Cord.

[113] Camfferman D., Moseley G.L., Gertz K., Pettet M.W., Jensen M.P. (2017). Waking EEG cortical markers of chronic pain and sleepiness. Pain Med..

[114] Zebhauser P.T., Hohn V.D., Ploner M. (2023). Resting-state electroencephalography and magnetoencephalography as biomarkers of chronic pain: A systematic review. Pain.

[115] Allison T., McCARTHY G., Wood C.C., Jones S.J. (1991). Potentials evoked in human and monkey cerebral cortex by stimulation of the median nerve: A review of scalp and intracranial recordings. Brain.

[116] Rossini P., Gigli G., Marciani M., Zarola F., Caramia M. (1987). Non-invasive evaluation of input-output characteristics of sensorimotor cerebral areas in healthy humans. Electroencephalogr. Clin. Neurophysiol./Evoked Potentials Sect..

[117] Funahashi S., Andreau J.M. (2013). Prefrontal cortex and neural mechanisms of executive function. J. Physiol..

[118] Haavik H., Anrig C., Plaugher G. (2022). The Contemporary Understanding of the Chiropractic Subluxation. Pediatric Chiropractic.

[119] Eippert F., Bingel U., Schoell E.D., Yacubian J., Klinger R., Lorenz J., Büchel C. (2009). Activation of the opioidergic descending pain control system underlies placebo analgesia. Neuron.

[120] Kong J., Loggia M.L., Zyloney C., Tu P., LaViolette P., Gollub R.L. (2010). Exploring the brain in pain: Activations, deactivations and their relation. Pain.

[121] Li W., Gong Y., Liu J., Guo Y., Tang H., Qin S., Zhao Y., Wang S., Xu Z., Chen B. (2021). Peripheral and central pathological mechanisms of chronic low back pain: A narrative review. J. Pain Res..

[122] De Ridder D., Adhia D., Vanneste S. (2021). The anatomy of pain and suffering in the brain and its clinical implications. Neurosci. Biobehav. Rev..

[123] Cohen S.P., Vase L., Hooten W.M. (2021). Chronic pain: An update on burden, best practices, and new advances. Lancet.

[124] Fitzcharles M.-A., Cohen S.P., Clauw D.J., Littlejohn G., Usui C., Häuser W. (2021). Nociplastic pain: Towards an understanding of prevalent pain conditions. Lancet.

[125] Ossipov M.H., Morimura K., Porreca F. (2014). Descending pain modulation and chronification of pain. Curr. Opin. Support. Palliat. Care.

[126] Rubinstein S.M., Terwee C.B., Assendelft W.J., de Boer M.R., van Tulder M.W. (2013). Spinal manipulative therapy for acute low back pain: An update of the cochrane review. Spine.

[127] Rubinstein S.M., De Zoete A., Van Middelkoop M., Assendelft W.J., De Boer M.R., Van Tulder M.W. (2019). Benefits and harms of spinal manipulative therapy for the treatment of chronic low back pain: Systematic review and meta-analysis of randomised controlled trials. BMJ.

[128] Furlan A.D., Yazdi F., Tsertsvadze A., Gross A., Van Tulder M., Santaguida L., Gagnier J., Ammendolia C., Dryden T., Doucette S. (2012). A systematic review and meta-analysis of efficacy, cost-effectiveness, and safety of selected complementary and alternative medicine for neck and low-back pain. Evid.-Based Complement. Altern. Med..

[129] Hidalgo B., Detrembleur C., Hall T., Mahaudens P., Nielens H. (2014). The efficacy of manual therapy and exercise for different stages of non-specific low back pain: An update of systematic reviews. J. Man. Manip. Ther..

[130] Paige N.M., Miake-Lye I.M., Booth M.S., Beroes J.M., Mardian A.S., Dougherty P., Branson R., Tang B., Morton S.C., Shekelle P.G. (2017). Association of spinal manipulative therapy with clinical benefit and harm for acute low back pain: Systematic review and meta-analysis. JAMA.

[131] Chou R., Côté P., Randhawa K., Torres P., Yu H., Nordin M., Hurwitz E.L., Haldeman S., Cedraschi C. (2018). The Global Spine Care Initiative: Applying evidence-based guidelines on the non-invasive management of back and neck pain to low-and middle-income communities. Eur. Spine J..

[132] Bailly F., Trouvin A.-P., Bercier S., Dadoun S., Deneuville J.-P., Faguer R., Fassier J.-B., Koleck M.l., Lassalle L., Le Vraux T. (2021). Clinical guidelines and care pathway for management of low back pain with or without radicular pain. Jt. Bone Spine.

[133] Kirkwood J., Allan G.M., Korownyk C.S., McCormack J., Garrison S., Thomas B., Ton J., Perry D., Kolber M.R., Dugré N. (2021). PEER simplified decision aid: Chronic back pain treatment options in primary care. Can. Fam. Physician.

[134] Yuan Q.-l., Guo T.-m., Liu L., Sun F., Zhang Y.-g. (2015). Traditional Chinese medicine for neck pain and low back pain: A systematic review and meta-analysis. PLoS ONE.

[135] Wu B., Yang L., Fu C., Jian G., Zhuo Y., Yao M., Xiong H. (2021). Efficacy and safety of acupuncture in treating acute low back pain: A systematic review and Bayesian network meta-analysis. Ann. Palliat. Med..

[136] Hawk C., Whalen W., Farabaugh R.J., Daniels C.J., Minkalis A.L., Taylor D.N., Anderson D., Anderson K., Crivelli L.S., Cark M. (2020). Best Practices for Chiropractic Management of Patients with Chronic Musculoskeletal Pain: A Clinical Practice Guideline. J. Altern. Complement. Med..

[137] Furman A.J., Meeker T.J., Rietschel J.C., Yoo S., Muthulingam J., Prokhorenko M., Keaser M.L., Goodman R.N., Mazaheri A., Seminowicz D.A. (2018). Cerebral peak alpha frequency predicts individual differences in pain sensitivity. Neuroimage.

[138] Furman A.J., Prokhorenko M., Keaser M.L., Zhang J., Chen S., Mazaheri A., Seminowicz D.A. (2020). Sensorimotor peak alpha frequency is a reliable biomarker of prolonged pain sensitivity. Cereb. Cortex.

[139] Hah J.M., Cramer E., Hilmoe H., Schmidt P., McCue R., Trafton J., Clay D., Sharifzadeh Y., Ruchelli G., Goodman S. (2019). Factors associated with acute pain estimation, postoperative pain resolution, opioid cessation, and recovery: Secondary analysis of a randomized clinical trial. JAMA Netw. Open.

[140] Millard S.K., Furman A.J., Kerr A., Seminowicz D.A., Gao F., Naidu B.V., Mazaheri A. (2022). Predicting postoperative pain in lung cancer patients using preoperative peak alpha frequency. Br. J. Anaesth..

[141] Michail G., Senkowski D., Niedeggen M., Keil J. (2021). Memory load alters perception-related neural oscillations during multisensory integration. J. Neurosci..

[142] Holt K.R., Haavik H., Lee A.C., Murphy B., Elley C.R. (2016). Effectiveness of Chiropractic Care to Improve Sensorimotor Function Associated With Falls Risk in Older People: A Randomized Controlled Trial. J. Manip. Physiol. Ther..

[143] Jamison J.R. (2005). Insomnia: Does chiropractic help?. J. Manip. Physiol. Ther..

[144] Adams J., Peng W., Cramer H., Sundberg T., Moore C., Amorin-Woods L., Sibbritt D., Lauche R. (2017). The Prevalence, patterns, and predictors of chiropractic use among US adults. Spine.

[145] Marino M.J., Langrell P.M. (1999). A longitudinal assessment of chiropractic care using a survey of self-rated health wellness & quality of life: A preliminary study. J. Vertebr. Subluxation Res..

[146] Boff T.A., Pasinato F., Ben Â.J., Bosmans J.E., van Tulder M., Carregaro R.L. (2020). Effectiveness of spinal manipulation and myofascial release compared with spinal manipulation alone on health-related outcomes in individuals with non-specific low back pain: Randomized controlled trial. Physiotherapy.

[147] Senna M.K., Machaly S.A. (2011). Does maintained spinal manipulation therapy for chronic nonspecific low back pain result in better long-term outcome?. Spine.

[148] Hays R.D., Spritzer K.L., Sherbourne C.D., Ryan G.W., Coulter I.D. (2019). Group and individual-level change on health-related quality of life in chiropractic patients with chronic low back or neck pain. Spine.

[149] Brandl F., Weise B., Mulej Bratec S., Jassim N., Hoffmann Ayala D., Bertram T., Ploner M., Sorg C. (2022). Common and specific large-scale brain changes in major depressive disorder, anxiety disorders, and chronic pain: A transdiagnostic multimodal meta-analysis of structural and functional MRI studies. Neuropsychopharmacology.

[150] Kim S., Lee J., Boone D. (2022). Protective and risk factors at the intersection of chronic pain, depression, anxiety, and somatic amplification: A latent profile approach. J. Pain Res..

[151] Humo M., Lu H., Yalcin I. (2019). The molecular neurobiology of chronic pain–induced depression. Cell Tissue Res..

[152] Vos T., Abajobir A.A., Abate K.H., Abbafati C., Abbas K.M., Abd-Allah F., Abdulkader R.S., Abdulle A.M., Abebo T.A., Abera S.F. (2017). Global, regional, and national incidence, prevalence, and years lived with disability for 328 diseases and injuries for 195 countries, 1990–2016: A systematic analysis for the Global Burden of Disease Study 2016. Lancet.

[153] Raffaeli W., Arnaudo E. (2017). Pain as a disease: An overview. J. Pain Res..

[154] Jackson T.P., Stabile V.S., McQueen K.K. (2014). The global burden of chronic pain. ASA Monitor.

[155] Kuner R., Flor H. (2016). Structural plasticity and reorganisation in chronic pain. Nat. Rev. Neurosci..

[156] Eklund A., Jensen I., Lohela-Karlsson M., Hagberg J., Leboeuf-Yde C., Kongsted A., Bodin L., Axén I. (2018). The Nordic Maintenance Care program: Effectiveness of chiropractic maintenance care versus symptom-guided treatment for recurrent and persistent low back pain—A pragmatic randomized controlled trial. PLoS ONE.

[157] Herman P.M., Edgington S.E., Ryan G.W., Coulter I.D. (2019). Prevalence and characteristics of chronic spinal pain patients with different hopes (treatment goals) for ongoing chiropractic care. J. Altern. Complement. Med..

[158] Herman P.M., Edgington S.E., Sorbero M.E., Hurwitz E.L., Goertz C.M., Coulter I.D. (2021). Visit frequency and outcomes for patients using ongoing chiropractic care for chronic low-back and neck pain: An observational longitudinal study. Pain Physician.

[159] Whedon J.M., Kizhakkeveettil A., Toler A.W., Bezdjian S., Rossi D., Uptmor S., MacKenzie T.A., Lurie J.D., Hurwitz E.L., Coulter I. (2022). Initial choice of spinal manipulation reduces escalation of care for chronic low back pain among older medicare beneficiaries. Spine.

[160] MacDonald D.A., Moseley G.L., Hodges P.W. (2006). The lumbar multifidus: Does the evidence support clinical beliefs?. Man. Ther..

[161] MacDonald D., Moseley G.L., Hodges P.W. (2009). Why do some patients keep hurting their back? Evidence of ongoing back muscle dysfunction during remission from recurrent back pain. Pain.

[162] Meier M.L., Vrana A., Schweinhardt P. (2018). Low Back Pain: The Potential Contribution of Supraspinal Motor Control and Proprioception. Neuroscientist.

[163] Pickar J.G., Sung P.S., Kang Y.M., Ge W. (2007). Response of lumbar paraspinal muscles spindles is greater to spinal manipulative loading compared with slower loading under length control. Spine J..

[164] Holt K.R. (2014). Effectiveness of Chiropractic Care in Improving Sensorimotor Function Associated with Falls Risk in Older People. Ph.D. Thesis.

[165] Morton D.L., Sandhu J.S., Jones A.K. (2016). Brain imaging of pain: State of the art. J. Pain Res..

[166] Hallez H., Vanrumste B., Grech R., Muscat J., De Clercq W., Vergult A., D’Asseler Y., Camilleri K.P., Fabri S.G., Van Huffel S. (2007). Review on solving the forward problem in EEG source analysis. J. Neuroeng. Rehabil..

[167] Peyron R., Frot M., Schneider F., Garcia-Larrea L., Mertens P., Barral F.-G., Sindou M., Laurent B., Mauguière F. (2002). Role of operculoinsular cortices in human pain processing: Converging evidence from PET, fMRI, dipole modeling, and intracerebral recordings of evoked potentials. Neuroimage.

[168] Mokhtari T., Tu Y., Hu L. (2019). Involvement of the hippocampus in chronic pain and depression. Brain Sci. Adv..

[169] Buckner R.L., DiNicola L.M. (2019). The brain’s default network: Updated anatomy, physiology and evolving insights. Nat. Rev. Neurosci..

