# Supplementary material for: Neuroplastic Responses to Chiropractic Care: Broad Impacts on Pain, Mood, Sleep, and Quality of Life
Source: Brain Sci. 2024 Nov 7;14(11):1124. doi: 10.3390/brainsci14111124 (PMC11592102; doi:10.3390/brainsci14111124)
Supplement: Supplementary file 1 [file brainsci-14-01124-s001.zip › Detailed discussion about EEG and SEPs Source Localisation Changes -Supplementary File S2.pdf]

Supplementary File S2. Detailed discussion about EEG and SEPs Source Localisation Changes

4.1. Resting state EEG source localisation

4.1.1. Resting state EEG PRE vs POST first session of chiropractic care

4.1.1.1. Alpha power increased functional connectivity between right Parahippocampal cortex and right Posterior Cingulate Cortex (R PCC) and Alpha power increased functional connectivity between the left Parahippocampal cortex (L ParaH) cortex and the right medial Orbitofrontal cortex (R MOF).

Alpha rhythms are known to play a key role in scene perception [1]. The PCC is important for many cognitive functions [2,3], including navigation and spatial memory [2,3] and for emotional regulation and awareness [4-7]. The MOF cortex is essential for processing information about one's own mental states, beliefs, intentions and desires for the future [4-7]. It plays a key role in introspection and the construction of a narrative sense of self [4-7], and is known to be well connected to the ParaH cortex which is vital for memory formation and retrieval [8-12], including the emotional modulation of memories [9,13-15]. Furthermore, the MOF has been shown to have reduced functional connectivity in depressed individuals [16]. Thus, the increased functional connectivity in Alpha power between the PCC and the ParaH cortex and the MOF and the ParaH straight after an adjustment most likely reflects a state of heightened introspection, or internally directed cognition and/or increased emotional and spatial awareness after the adjustment. This is supported by previous research that has, for example, shown that straight after spinal adjustments there is improved proprioception of the spine [17], and extremities such as the elbow joint [18]. Other studies have shown that the presence of spinal dysfunction is associated with less accurate and slower multisensory integration of sound and visual information [19] that would impact processing and integration of object recognition and scene perception. Furthermore, a clinical trial showed improved multisensory integration of sound and visual information in a group of older adults following 12 weeks of chiropractic care [20]. These findings align with our between-group spectral analysis, which highlighted an increase in Alpha-band activity in the central regions of the brain. Alpha power is also commonly found to be lower in individuals with chronic pain, localized in the insula and particularly in the frontal lobes, when compared with healthy controls [21-24]. Therefore, it's possible that the Alpha power frequency changes found in this study also reflect changes in pain perception. However, some argue that changes in Alpha power may not be exclusively found in pain patients, as it is also found in attention deficit hyperactivity disorder, depression, cognitive function, and age [24]. It is therefore most likely that the change in Alpha activity in our study reflects a combination of changes in processing internal body signals, emotional processing and spatial awareness after the first adjustment session, resulting in better QOL, and sleep.

4.1.2. Resting state EEG PRE vs POST-4-weeks of chiropractic care

4.1.2.1. Alpha power increased functional connectivity between the right Parahippocampal cortex (R ParaH) cortex and the right Precuneus (R Precun).

The ParaH cortex is vital for memory formation and retrieval [8-15] and is also essential for spatial navigation, object recognition and scene perception [9]. The Precun is highly involved in self-awareness, self-referential processing and self-reflection [25,26] and is also essential for visuospatial integration [27]. In particular, the anterior Precun is known as a key hub for where the brain processes and becomes aware of its bodily self, i.e., the body schema [28,29]. So much so that some argue that the anterior Precun can be viewed as a ''convergence zone'' for the core body schema that is continuously (and subconsciously) updated as the organism moves within the environment [28,29]. Thus, it would appear that this increased functional connectivity between the ParaH and Precun in the current study participants resting EEG reflects their brains updating their body schema and/or external world schema. This makes logical sense as the adjustments would be 'bombarding' the brain with paraspinal proprioceptive signals that would influence the body schema, and the body schema would influence the external world schema in turn. As these functional connectivity changes were between the baseline recording and post four weeks of chiropractic care it is also possible that this change reflects a difference in how they are reflecting on themselves and their everyday experiences over time. This reflection after four weeks of care appears now to be more positive, since they also reported experiencing less pain, less fatigue, less anxiety and less depression and improved sleep after the four weeks of chiropractic care.

4.1.2.2. Alpha power increased in functional connectivity between right lateral Orbitofrontal (R LOF) and left Isthmus Cingulate cortex (L ICC)

The Orbitofrontal cortex participates in the executive control of information processing and behavioral expression by inhibiting neural activity associated with irrelevant, unwanted, or uncomfortable (e.g. painful) information, sensations, or actions [16,30-32]. The LOF is important for assessing the emotional significance of stimuli which impacts our decision making based on the emotional value of those stimuli [16,32]. The LOF is also known to facilitate selective attention by controlling (inhibiting the processing of) the interference of irrelevant emotional information in the environment [31]. Similarly, the LOF along with the ventrolateral PFC, are also known to regulate and inhibit emotions and their expression in a top down manner [16,31-35]. The ICC is implicated in self-referential processing and introspection [36,37]. Therefore, this change in functional connectivity likely reflects that 4 weeks of chiropractic care resulted in changes in the way participant's brains assessed the emotional significance of sensory stimuli. The participants may now no longer connect proprioceptive information coming from the spine with painful or unpleasant feelings. Furthermore, these participants appear to have altered their own emotional evaluation of internal thoughts about their emotional states and their emotional control since they reported improvements in fatigue, anxiety and depression symptoms over the four weeks of care.

4.1.2.3. Alpha power increased in functional connectivity between the left rostral Anterior Cingulate Cortex (L RACC) cortex and right medial Orbitofrontal (R MOF) and Alpha power increase in functional

connectivity between the left rostral Anterior Cingulate Cortex (L RACC) and left medial Orbitofrontal (L MOF)

As discussed earlier, the MOF cortex plays a key role in one's own understanding of our mental states, beliefs, intentions and desires [4-7] and has reduced functional connectivity in those who suffer from depression [16]. The ACC, which is part of the DMN is also a key hub of the Salience network [38,39] and has two subdivision (rostral and caudal parts). The RACC has strong limbic connections, orbitofrontal connections, anterior insula connections (relevant for interoception), periaqueductal gray (PAG) connections (relevant for descending pain control), nucleus accumbens connections (relevant for reward regulation) and hypothalamus connections (relevant for ANS regulation) [40]. The RACC is particularly involved in recognizing and understanding one's own emotions [41] and also plays a key role in the descending pain inhibitory pathway [42-44]. The Salience network and DMN are also known to have abnormal functional connectivity in people with chronic pain, depression and anxiety [45]. Thus, considering the improvements in anxiety and depression along with a reduction in pain this increase in Alpha power functional connectivity between the RACC and both right and left MOF cortex probably reflects improved functional connectivity between the participants salience network and their DMNs. This increased functional connectivity likely reflects the more positive way in which these participants are constructing a sense of self and how they are understanding their own mental states that, after four weeks of chiropractic care, includes significantly less depressive, anxious and pain symptoms. The reduction in pain symptoms over the four weeks may be due to an increase in descending pain inhibition, following the increased activity in the RACC. As the current studies analysis only explored functional connectivity within the DMN, this finding highlights the need to re-evaluate this studies data to also explore changes in within the Salience Network.

4.2. SEPs source localisation

4.2.1. SEPs PRE vs POST first session of chiropractic care

4.2.1.1. Beta power decreased in functional connectivity between right medial Orbitofrontal (R MOF) cortex and left Precuneus (L Precun)

The Precun is highly involved in self-awareness, self-referential processing and self-reflection, including learning and consolidating everyday experiences [25,26]. As mentioned earlier, it is also interesting to note that the anterior Precun is where the brain processes and becomes aware of its bodily self, thus it is essential for the construction of the brain's body schema [28,29]. The MOF cortex plays a key role in processing information about one's own mental states, beliefs, intentions and desires, introspection, and in constructing a sense of self [4-7]. Thus, this change in functional connectivity between MOF cortex and Precun may reflect the changes in body schema from the spinal adjustments, also altering the participants mental states, introspection and their constructed sense of self.

4.2.1.2. Beta power decreased in functional connectivity between left lateral Orbitofrontal (L LOF) cortex and left Posterior Cingulate cortex (L PCC)

The LOF is important for assessing and responding to the emotional significance of stimuli [16,32] and is also known to facilitate selective attention by inhibiting the processing of irrelevant emotional information in the environment [31]. Similarly, the LOF along with the ventrolateral PFC, are known to regulate and inhibit emotions and their expression [16,31-35]. It has also been shown that people suffering with depression have an increased functional connectivity between the Orbitofrontal cortex, the Precuneus and/or the PCC [16,46-48], which is thought to reflect excessive negative ruminations about oneself that occurs in depression [16,46-48]. The current study revealed that over the four-week intervention period, the participants in the chiropractic group reported decreased depression symptoms. It is possible that this observed decreased functional connectivity in beta activity between the LOF and PCC straight after the first session of chiropractic care may have altered the way the subjects regulated and inhibited emotions in a top-down manner that over the four weeks resulted in less feelings of depression.

4.2.1.3. Alpha power increased in functional connectivity between the right Isthmus Cingulate cortex (R ICC) and left medial Orbitofrontal (L MOF) cortex

Both the ICC [36,37] and MOF [4-7] are implicated in self-referential processing and introspection. This may therefore suggest that this observed increase in functional connectivity might reflect heightened self-awareness and introspection, i.e., awareness and thinking of one's internal state straight after the first adjustment session.

4.2.1.4. Alpha power increased in functional connectivity between the left Parahippocampal (L ParaH) cortex and left rostral Anterior Cingulate cortex (L RACC)

As mentioned earlier, the ParaH cortex is vital for memory formation and retrieval [8-12] including the emotional modulation of memories [9,13-15]. It is also essential for spatial navigation, object recognition and scene perception [8-12], and for the construction of mental scenes while recalling autobiographical experiences and imagining future events [8-12]. As also mentioned earlier, the ACC included in the DMN is also a key hub of the Salience network [38,39]. Thus, the increased Alpha power functional connectivity observed following adjustments could reflect changes in interpretation of the internal environment (interoception), as well as the external world (exteroception), thus enabling the participants brain's to more accurately perceive what is occurring in their bodies and in the scene around them (e.g., object recognition). Support for this notion is that previous research has shown chiropractic adjustments improve the accuracy and speed of visual and auditory information processing [20], as well as improvements in proprioception following adjustments [17,18,20], as well as faster and more accurate mental rotation [49]. It is likely that the adjustment is a highly salient signal to the brain, which as mentioned earlier, warrants a reassessment of the EEG and SEPs data to also explore for possible alterations in Salience Network activity. For example, it is possible the change in Alpha power functional connectivity between the ParaH cortex and RACC reflects the activation of the descending pain control network, as these participants also reported decreased pain at post-4-weeks intervention. Both the RACC and the ParaH area are known to be involved in the descending pain inhibitory pathway [42-44]. This is particularly interesting, because it is well known

that the descending modulatory pain pathway is malfunctional in chronic pain patients [42,50-53]. In addition, the malfunction of the descending modulatory pain pathway has been implicated in the 'chronification' of acute to chronic pain [54]. Our study provides some support for the notion that part of the mechanisms of chiropractic care is that it might improve the descending modulatory pain pathway in chronic low back pain patients, and this may be why it has been found to be clinically effective for both acute and chronic low back pain problems and is a recommended option in relevant clinical guidelines [55-65].

4.2.2. SEPs PRE vs POST-4-weeks of chiropractic care

4.2.2.1. Alpha power decreased functional connectivity between right Parahippocampal (R Para H) cortex and left lateral Orbitofrontal (L LOF) cortex and Beta power decreased functional connectivity between right lateral Orbitofrontal (R LOF) cortex and left Parahippocampal (L ParaH) cortex

As noted earlier, the Orbitofrontal cortex participates in the executive control of information processing and behavioral expression by inhibiting neural activity associated with irrelevant, unwanted, or uncomfortable (e.g. painful) information, sensations, or actions [16,30-32]. Not only does the LOF cortex impact decision making based on the assessment of the emotional significance of stimuli [16,32], but it also impacts selective attention by inhibiting the processing of irrelevant emotional information in the environment [31]. Thus the LOF cortex has a strong influence over our emotions and their expression in a top down manner [16,31-35]. The ParaH cortex is, as previously discussed, vital for memory formation and retrieval [8-12], emotional modulation of memories [9,13-15], spatial navigation, object recognition, scene perception [8-12]. Thus, the decrease in both Alpha and Beta power functional connectivity between the LOF and L ParaH area likely reflects changes to self-referential processing and emotional evaluation of internal and external stimuli, and regulation of emotions and memories over the 4 weeks of chiropractic care. The HVLA adjustments of vertebral subluxations over the four weeks has likely altered the sensory stimuli coming from the spine, and this appears to have changed the way the LOF cortex and ParaH cortex are assessing the emotional significance of that altered spinal sensory input, thus altering the way executive control functions operate, i.e., now, after the adjustments there are less uncomfortable and painful sensations associated with their spinal sensory information. Their internal narrative sense of self also appears to have been altered over the four weeks of care, with changes in the emotional processing associated with internal and external stimuli which is influencing how they perceive themselves and the world around them, reflected by the reported decrease in fatigue, depression, anxiety and pain.

4.2.2.2. Alpha power increased functional connectivity between the left Isthmus Cingulate cortex (L ICC) and the right Posterior Cingulate cortex (R PCC) and Alpha power decreased functional connectivity between the left Posterior Cingulate cortex (L PCC) and right Posterior Cingulate cortex (R PCC).

The PCC is important for many cognitive functions [2,3], including navigation and spatial memory [2,3], and emotional regulation and

awareness [4-7]. The PCC is particularly active during internally focused, self-referential processing, and is less active during externally focused processing [66], and is known to be implicated in depression [67,68]. The ICC, is the tail end of the PCC and is also implicated in introspection and self-referential processing, including the construction of the narrative sense of self and autobiographical memory formation and retrieval [36,37]. Research has shown that depressed individuals have an asymmetry in the thickness of their PCC, with greater cortical thickness in the left compared to right hemisphere [67,68]. In particular, depressed individuals with higher somatic symptoms such as sleep disturbance, appetite disturbance, fatigue or loss of energy have greater asymmetry in the thickness of their PCC [67]. In light of the decrease in depression symptoms in the current study, along with positive changes in sleep and fatigue, it is possible that the changes in functional connectivity in Alpha power between the L PCC (including ICC) and R PCC reflects the participants altered construction of their narrative sense of self (self-referential processing) in a manner that has resulted in less symptoms of depression that has led to improved energy (i.e. less fatigue) and sleep.

4.2.2.3. Beta power decreased functional connectivity between the right rostral Anterior Cingulate cortex (R RACC) and left Precuneus (L Precun)

The rostral division of the ACC is highly involved in interoception and recognizing and understanding one's own emotions [41]. It is also essential for assessing the salience of emotional and motivational information and regulating our emotional responses [69,70]. The RACC also influences the descending pain control system through its strong connections to the PAG [40]. The Precun is a key hub of the DMN and is highly involved in self-awareness, self-referential processing and self-reflection, including learning and consolidating everyday experiences [25,26]. Abnormal connectivity has been found between the salience network and DMNs in major depression disorder [45]. Knowing this, it seems likely that the decrease in functional connectivity between R RACC and L Precun could be a reflection of deceased connectivity between the Salience Network and the DMN, resulting in decreased pain as well as less depression and anxiety symptoms after 4 weeks of chiropractic care. It is therefore imperative to re-analyse the data from this study to also explore changes in Salience Network activity.

References:

1. Stecher, R.; Kaiser, D. Representations of imaginary scenes and their properties in cortical alpha activity. *Scientific Reports* **2024**, *14*, 12796.
2. Spreng, R.N.; Mar, R.A.; Kim, A.S. The common neural basis of autobiographical memory, prospection, navigation, theory of mind, and the default mode: a quantitative meta-analysis. *Journal of cognitive neuroscience* **2009**, *21*, 489-510.
3. Foster, B.L.; Koslov, S.R.; Aponik-Gremillion, L.; Monko, M.E.; Hayden, B.Y.; Heilbronner, S.R. A tripartite view of the posterior cingulate cortex. *Nature Reviews Neuroscience* **2023**, *24*, 173-189.
4. Moscovitch, M.; Cabeza, R.; Winocur, G.; Nadel, L. Episodic memory and beyond: the hippocampus and neocortex in transformation. *Annual review of psychology* **2016**, *67*, 105-134.
5. Menon, V. 20 years of the default mode network: A review and synthesis. *Neuron* **2023**.

6. Davey, C.G.; Pujol, J.; Harrison, B.J. Mapping the self in the brain's default mode network. *NeuroImage* **2016**, *132*, 390-397.
7. Whitfield-Gabrieli, S.; Moran, J.M.; Nieto-Castañón, A.; Triantafyllou, C.; Saxe, R.; Gabrieli, J.D. Associations and dissociations between default and self-reference networks in the human brain. *Neuroimage* **2011**, *55*, 225-232.
8. Peng, X.; Burwell, R.D. Beyond the hippocampus: the role of parahippocampal-prefrontal communication in context-modulated behavior. *Neurobiology of learning and memory* **2021**, *185*, 107520.
9. Aminoff, E.M.; Kveraga, K.; Bar, M. The role of the parahippocampal cortex in cognition. *Trends in cognitive sciences* **2013**, *17*, 379-390.
10. Andrews-Hanna, J.R.; Saxe, R.; Yarkoni, T. Contributions of episodic retrieval and mentalizing to autobiographical thought: Evidence from functional neuroimaging, resting-state connectivity, and fMRI meta-analyses. *NeuroImage* **2014**, *91*, 324-335, doi:https://doi.org/10.1016/j.neuroimage.2014.01.032.
11. Andrews-Hanna, J.R.; Reidler, J.S.; Sepulcre, J.; Poulin, R.; Buckner, R.L. Functional-anatomic fractionation of the brain's default network. *Neuron* **2010**, *65*, 550-562.
12. Gerin, M.I.; Viding, E.; Herringa, R.J.; Russell, J.D.; McCrory, E.J. A systematic review of childhood maltreatment and resting state functional connectivity. *Developmental Cognitive Neuroscience* **2023**, *64*, 101322, doi:https://doi.org/10.1016/j.dcn.2023.101322.
13. Smith, A.P.; Henson, R.N.; Dolan, R.J.; Rugg, M.D. fMRI correlates of the episodic retrieval of emotional contexts. *Neuroimage* **2004**, *22*, 868-878.
14. Gosselin, N.; Samson, S.; Adolphs, R.; Noulhiane, M.; Roy, M.; Hasboun, D.; Baulac, M.; Peretz, I. Emotional responses to unpleasant music correlates with damage to the parahippocampal cortex. *Brain* **2006**, *129*, 2585-2592.
15. Mitterschiffthaler, M.T.; Fu, C.H.; Dalton, J.A.; Andrew, C.M.; Williams, S.C. A functional MRI study of happy and sad affective states induced by classical music. *Human brain mapping* **2007**, *28*, 1150-1162.
16. Rolls, E.T.; Cheng, W.; Feng, J. The orbitofrontal cortex: reward, emotion and depression. *Brain communications* **2020**, *2*, fcaa196.
17. Palmgren, P.J.; Sandström, P.J.; Lundqvist, F.J.; Heikkilä, H. Improvement after chiropractic care in cervicocephalic kinesthetic sensibility and subjective pain intensity in patients with nontraumatic chronic neck pain. *Journal of manipulative and physiological therapeutics* **2006**, *29*, 100-106.
18. Haavik, H.; Murphy, B. Subclinical neck pain and the effects of cervical manipulation on elbow joint position sense. *Journal of manipulative and physiological therapeutics* **2011**, *34*, 88-97.
19. Farid, B.; Yielder, P.; Holmes, M.; Haavik, H.; Murphy, B.A. Association of subclinical neck pain with altered multisensory integration at baseline and 4-week follow-up relative to asymptomatic controls. *Journal of manipulative and physiological therapeutics* **2018**, *41*, 81-91.
20. Holt, K.R.; Haavik, H.; Lee, A.C.; Murphy, B.; Elley, C.R. Effectiveness of Chiropractic Care to Improve Sensorimotor Function Associated With Falls Risk in Older People: A Randomized Controlled Trial. *J Manipulative Physiol Ther* **2016**, 10.1016/j.jmpt.2016.02.003, doi:10.1016/j.jmpt.2016.02.003.
21. Stern, J.; Jeanmonod, D.; Sarnthein, J. Persistent EEG overactivation in the cortical pain matrix of neurogenic pain patients. *Neuroimage* **2006**, *31*, 721-731.
22. Jensen, M.; Sherlin, L.; Gertz, K.; Braden, A.; Kupper, A.; Gianas, A.; Howe, J.; Hakimian, S. Brain EEG activity correlates of chronic pain in persons with spinal cord injury: clinical implications. *Spinal Cord* **2013**, *51*, 55-58.

23. Camfferman, D.; Moseley, G.L.; Gertz, K.; Pettet, M.W.; Jensen, M.P. Waking EEG cortical markers of chronic pain and sleepiness. *Pain Medicine* **2017**, *18*, 1921-1931.
24. Zebhauser, P.T.; Hohn, V.D.; Ploner, M. Resting-state electroencephalography and magnetoencephalography as biomarkers of chronic pain: a systematic review. *Pain* **2023**, *164*, 1200-1221, doi:10.1097/j.pain.0000000000002825.
25. Cavanna, A.E. The precuneus and consciousness. *CNS spectrums* **2007**, *12*, 545-552.
26. Dadario, N.B.; Sughrue, M.E. The functional role of the precuneus. *Brain* **2023**, *146*, 3598-3607, doi:10.1093/brain/awad181.
27. Bruner, E.; Preuss, T.M.; Chen, X.; Rilling, J.K. Evidence for expansion of the precuneus in human evolution. *Brain Structure and Function* **2017**, *222*, 1053-1060.
28. Lyu, D.; Stieger, J.R.; Xin, C.; Ma, E.; Lusk, Z.; Aparicio, M.K.; Werbaneth, K.; Perry, C.M.; Deisseroth, K.; Buch, V. Causal evidence for the processing of bodily self in the anterior precuneus. *Neuron* **2023**, *111*, 2502-2512. e2504.
29. Berlucchi, G.; Aglioti, S. The body in the brain: neural bases of corporeal awareness. *Trends in neurosciences* **1997**, *20*, 560-564.
30. Shimamura, A.P. The role of the prefrontal cortex in dynamic filtering. *Psychobiology* **2000**, *28*, 207-218.
31. Hooker, C.I.; Knight, R.T. The role of lateral orbitofrontal cortex in the inhibitory control of emotion. *The orbitofrontal cortex* **2006**, *307*, 1-18.
32. Rolls, E.T. The cingulate cortex and limbic systems for action, emotion, and memory. *Handbook of clinical neurology* **2019**, *166*, 23-37.
33. Ochsner, K.N.; Bunge, S.A.; Gross, J.J.; Gabrieli, J.D. Rethinking feelings: an FMRI study of the cognitive regulation of emotion. *Journal of cognitive neuroscience* **2002**, *14*, 1215-1229.
34. Ochsner, K.N.; Ray, R.D.; Cooper, J.C.; Robertson, E.R.; Chopra, S.; Gabrieli, J.D.; Gross, J.J. For better or for worse: neural systems supporting the cognitive down-and up-regulation of negative emotion. *Neuroimage* **2004**, *23*, 483-499.
35. Phan, K.L.; Fitzgerald, D.A.; Nathan, P.J.; Moore, G.J.; Uhde, T.W.; Tancer, M.E. Neural substrates for voluntary suppression of negative affect: a functional magnetic resonance imaging study. *Biological psychiatry* **2005**, *57*, 210-219.
36. Northoff, G.; Bermpohl, F. Cortical midline structures and the self. *Trends in cognitive sciences* **2004**, *8*, 102-107.
37. Vogeley, K.; Bussfeld, P.; Newen, A.; Herrmann, S.; Happé, F.; Falkai, P.; Maier, W.; Shah, N.J.; Fink, G.R.; Zilles, K. Mind Reading: Neural Mechanisms of Theory of Mind and Self-Perspective. *NeuroImage* **2001**, *14*, 170-181, doi:https://doi.org/10.1006/nimg.2001.0789.
38. Seeley, W.W. The salience network: a neural system for perceiving and responding to homeostatic demands. *Journal of Neuroscience* **2019**, *39*, 9878-9882.
39. Schimmelpfennig, J.; Topczewski, J.; Zajkowski, W.; Jankowiak-Siuda, K. The role of the salience network in cognitive and affective deficits. *Frontiers in human neuroscience* **2023**, *17*, 1133367.
40. Washington, S.D.; VanMeter, J.W. Anterior-posterior connectivity within the default mode network increases during maturation. *International journal of medical and biological frontiers* **2015**, *21*, 207.
41. Smith, R.; Ahern, G.L.; Lane, R.D. The role of anterior and midcingulate cortex in emotional awareness: A domain-general processing perspective. *Handbook of clinical neurology* **2019**, *166*, 89-101.
42. De Ridder, D.; Vanneste, S.; Smith, M.; Adhia, D. Pain and the triple network model. *Frontiers in neurology* **2022**, *13*, 757241.

43. Eippert, F.; Bingel, U.; Schoell, E.D.; Yacubian, J.; Klinger, R.; Lorenz, J.; Büchel, C. Activation of the opioidergic descending pain control system underlies placebo analgesia. *Neuron* **2009**, *63*, 533-543.
44. Kong, J.; Loggia, M.L.; Zyloney, C.; Tu, P.; LaViolette, P.; Gollub, R.L. Exploring the brain in pain: activations, deactivations and their relation. *Pain* **2010**, *148*, 257-267.
45. Brandl, F.; Weise, B.; Mulej Bratec, S.; Jassim, N.; Hoffmann Ayala, D.; Bertram, T.; Ploner, M.; Sorg, C. Common and specific large-scale brain changes in major depressive disorder, anxiety disorders, and chronic pain: a transdiagnostic multimodal meta-analysis of structural and functional MRI studies. *Neuropsychopharmacology* **2022**, *47*, 1071-1080.
46. Cheng, W.; Rolls, E.T.; Qiu, J.; Xie, X.; Wei, D.; Huang, C.-C.; Yang, A.C.; Tsai, S.-J.; Li, Q.; Meng, J. Increased functional connectivity of the posterior cingulate cortex with the lateral orbitofrontal cortex in depression. *Translational Psychiatry* **2018**, *8*, 90.
47. Kandilarova, S.; Stoyanov, D.; Aryutova, K.; Paunova, R.; Mantarkov, M.; Mitrev, I.; Todeva-Radneva, A.; Specht, K. Effective connectivity between the orbitofrontal cortex and the precuneus differentiates major psychiatric disorders: Results from a transdiagnostic spectral DCM study. *CNS & Neurological Disorders-Drug Targets (Formerly Current Drug Targets-CNS & Neurological Disorders)* **2023**, *22*, 180-190.
48. Zhu, Z.; Wang, Y.; Lau, W.K.; Wei, X.; Liu, Y.; Huang, R.; Zhang, R. Hyperconnectivity between the posterior cingulate and middle frontal and temporal gyrus in depression: Based on functional connectivity meta-analyses. *Brain Imaging and Behavior* **2022**, *16*, 1538-1551.
49. Kelly, D.D.; Murphy, B.A.; Backhouse, D.P. Use of a mental rotation reaction-time paradigm to measure the effects of upper cervical adjustments on cortical processing: a pilot study. *Journal of manipulative and physiological therapeutics* **2000**, *23*, 246-251.
50. Li, W.; Gong, Y.; Liu, J.; Guo, Y.; Tang, H.; Qin, S.; Zhao, Y.; Wang, S.; Xu, Z.; Chen, B. Peripheral and central pathological mechanisms of chronic low back pain: a narrative review. *JPR* **2021**, 1483-1494.
51. De Ridder, D.; Adhia, D.; Vanneste, S. The anatomy of pain and suffering in the brain and its clinical implications. *Neuroscience & Biobehavioral Reviews* **2021**, *130*, 125-146.
52. Cohen, S.P.; Vase, L.; Hooten, W.M. Chronic pain: an update on burden, best practices, and new advances. *The Lancet* **2021**, *397*, 2082-2097.
53. Fitzcharles, M.-A.; Cohen, S.P.; Clauw, D.J.; Littlejohn, G.; Usui, C.; Häuser, W. Nociplastic pain: towards an understanding of prevalent pain conditions. *The Lancet* **2021**, *397*, 2098-2110.
54. Ossipov, M.H.; Morimura, K.; Porreca, F. Descending pain modulation and chronification of pain. *Current opinion in supportive and palliative care* **2014**, *8*, 143-151.
55. Rubinstein, S.M.; Terwee, C.B.; Assendelft, W.J.; de Boer, M.R.; van Tulder, M.W. Spinal manipulative therapy for acute low back pain: an update of the cochrane review. *Spine* **2013**, *38*, E158-E177.
56. Rubinstein, S.M.; De Zoete, A.; Van Middelkoop, M.; Assendelft, W.J.; De Boer, M.R.; Van Tulder, M.W. Benefits and harms of spinal manipulative therapy for the treatment of chronic low back pain: systematic review and meta-analysis of randomised controlled trials. *bmj* **2019**, *364*, l689.
57. Furlan, A.D.; Yazdi, F.; Tsertsvadze, A.; Gross, A.; Van Tulder, M.; Santaguida, L.; Gagnier, J.; Ammendolia, C.; Dryden, T.; Doucette, S. A systematic review and meta-analysis of efficacy, cost-effectiveness, and safety of selected complementary and alternative medicine for neck and low-back pain. *Evidence-Based Complementary and Alternative Medicine* **2012**, *2012*, 953139.

58. Hidalgo, B.; Detrembleur, C.; Hall, T.; Mahaudens, P.; Nielens, H. The efficacy of manual therapy and exercise for different stages of non-specific low back pain: an update of systematic reviews. *Journal of Manual & Manipulative Therapy* **2014**, *22*, 59-74.
59. Paige, N.M.; Miake-Lye, I.M.; Booth, M.S.; Beroes, J.M.; Mardian, A.S.; Dougherty, P.; Branson, R.; Tang, B.; Morton, S.C.; Shekelle, P.G. Association of spinal manipulative therapy with clinical benefit and harm for acute low back pain: systematic review and meta-analysis. *Jama* **2017**, *317*, 1451-1460.
60. Chou, R.; Côté, P.; Randhawa, K.; Torres, P.; Yu, H.; Nordin, M.; Hurwitz, E.L.; Haldeman, S.; Cedraschi, C. The Global Spine Care Initiative: applying evidence-based guidelines on the non-invasive management of back and neck pain to low-and middle-income communities. *European Spine Journal* **2018**, *27*, 851-860.
61. Bailly, F.; Trouvin, A.-P.; Bercier, S.; Dadoun, S.; Deneuville, J.-P.; Faguer, R.; Fassier, J.-B.; Koleck, M.l.; Lassalle, L.; Le Vraux, T. Clinical guidelines and care pathway for management of low back pain with or without radicular pain. *Joint Bone Spine* **2021**, *88*, 105227.
62. Kirkwood, J.; Allan, G.M.; Korownyk, C.S.; McCormack, J.; Garrison, S.; Thomas, B.; Ton, J.; Perry, D.; Kolber, M.R.; Dugré, N. PEER simplified decision aid: chronic back pain treatment options in primary care. *Canadian Family Physician* **2021**, *67*, 31-34.
63. Yuan, Q.-l.; Guo, T.-m.; Liu, L.; Sun, F.; Zhang, Y.-g. Traditional Chinese medicine for neck pain and low back pain: a systematic review and meta-analysis. *PloS one* **2015**, *10*, e0117146.
64. Wu, B.; Yang, L.; Fu, C.; Jian, G.; Zhuo, Y.; Yao, M.; Xiong, H. Efficacy and safety of acupuncture in treating acute low back pain: a systematic review and Bayesian network meta-analysis. *Annals of Palliative Medicine* **2021**, *10*, 6156167-6156167.
65. Hawk, C.; Whalen, W.; Farabaugh, R.J.; Daniels, C.J.; Minkalis, A.L.; Taylor, D.N.; Anderson, D.; Anderson, K.; Crivelli, L.S.; Cark, M., et al. Best Practices for Chiropractic Management of Patients with Chronic Musculoskeletal Pain: A Clinical Practice Guideline. *J Altern Complement Med* **2020**, *26*, 884-901, doi:10.1089/acm.2020.0181.
66. Buckner, R.L.; Carroll, D.C. Self-projection and the brain. *Trends in cognitive sciences* **2007**, *11*, 49-57.
67. Dotson, V.M.; Taiwo, Z.; Minto, L.R.; Bogoian, H.R.; Gradone, A.M. Orbitofrontal and Cingulate Thickness Asymmetry Associated with Depressive Symptom Dimensions. *Cognitive, Affective, & Behavioral Neuroscience* **2021**, *21*, 1297-1305, doi:10.3758/s13415-021-00923-8.
68. Philip van Eijndhoven, M.D., Ph.D. ,; Guido van Wingen, Ph.D. ,; Maartje Katzenbauer, M.D. ,; Wouter Groen, M.D., Ph.D. ,; Ralf Tepest, Ph.D. ,; Guillen Fernández, M.D., Ph.D. ,; Jan Buitelaar, M.D., Ph.D. , and; Indira Tendolkar, M.D., Ph.D. Paralimbic Cortical Thickness in First-Episode Depression: Evidence for Trait-Related Differences in Mood Regulation. *American Journal of Psychiatry* **2013**, *170*, 1477-1486, doi:10.1176/appi.ajp.2013.12121504.
69. Devinsky, O.; Morrell, M.J.; Vogt, B.A. Contributions of anterior cingulate cortex to behaviour. *Brain* **1995**, *118*, 279-306.
70. Whalen, P.J.; Bush, G.; McNally, R.J.; Wilhelm, S.; McInerney, S.C.; Jenike, M.A.; Rauch, S.L. The emotional counting Stroop paradigm: a functional magnetic resonance imaging probe of the anterior cingulate affective division. *Biological psychiatry* **1998**, *44*, 1219-1228.
