# Supplementary material for: Neuroplastic Responses to Chiropractic Care: Broad Impacts on Pain, Mood, Sleep, and Quality of Life
Source: Brain Sci. 2024 Nov 7;14(11):1124. doi: 10.3390/brainsci14111124 (PMC11592102; doi:10.3390/brainsci14111124)
Supplement: Supplementary file 1 [file brainsci-14-01124-s001.zip › PROMIS-Supplementary File S1.pdf]

Neuroplastic Responses to Chiropractic Care: Supplementary File for PROMIS

Usman Rashid

22/07/2024

Contents

1 Data Visualisations . . . **2**
1.1 PROMIS . . . 2
1.2 PROMIS (Total) . . . 2

2 Statistical Comparison of Groups . . . **4**
2.1 PROMIS . . . 4
2.1.1 Model . . . 4
2.1.2 Diagnostics . . . 4
2.1.3 Analysis of Deviance . . . 4
2.1.4 Variation across Componenets . . . 5
2.1.5 Between Group Statistics . . . 5
2.1.6 Within Group Statistics . . . 5
2.2 PROMIS (Total) . . . 7
2.2.1 Model . . . 7
2.2.2 Diagnostics . . . 7
2.2.3 Analysis of Deviance . . . 7
2.2.4 Between Group Statistics . . . 7
2.2.5 Within Group Statistics . . . 8

1 Data Visualisations

1.1 PROMIS

```
## Warning: Removed 26 rows containing non-finite values (`stat_boxplot()`).

## Warning: Removed 26 rows containing missing values (`geom_point()`).
```

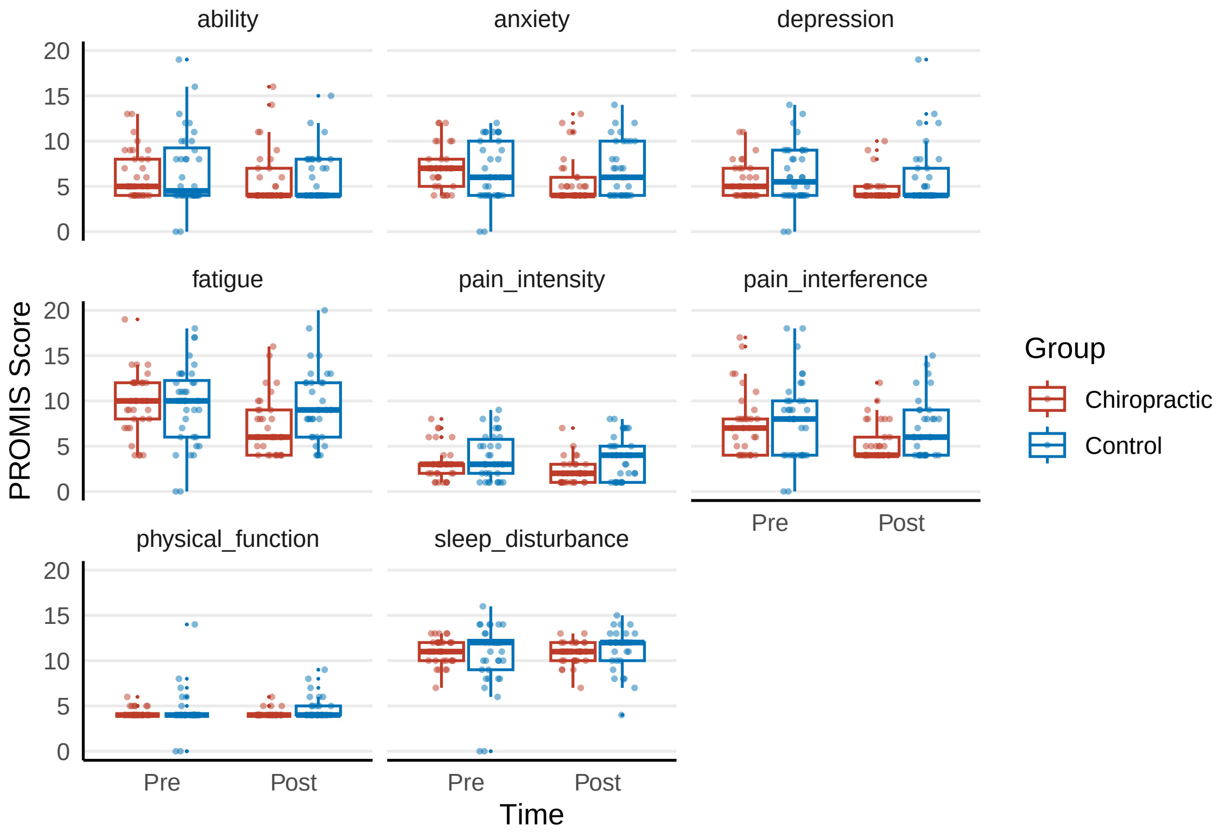

1.2 PROMIS (Total)

```
## Warning: Removed 4 rows containing non-finite values (`stat_boxplot()`).

## Warning: Removed 4 rows containing missing values (`geom_point()`).
```

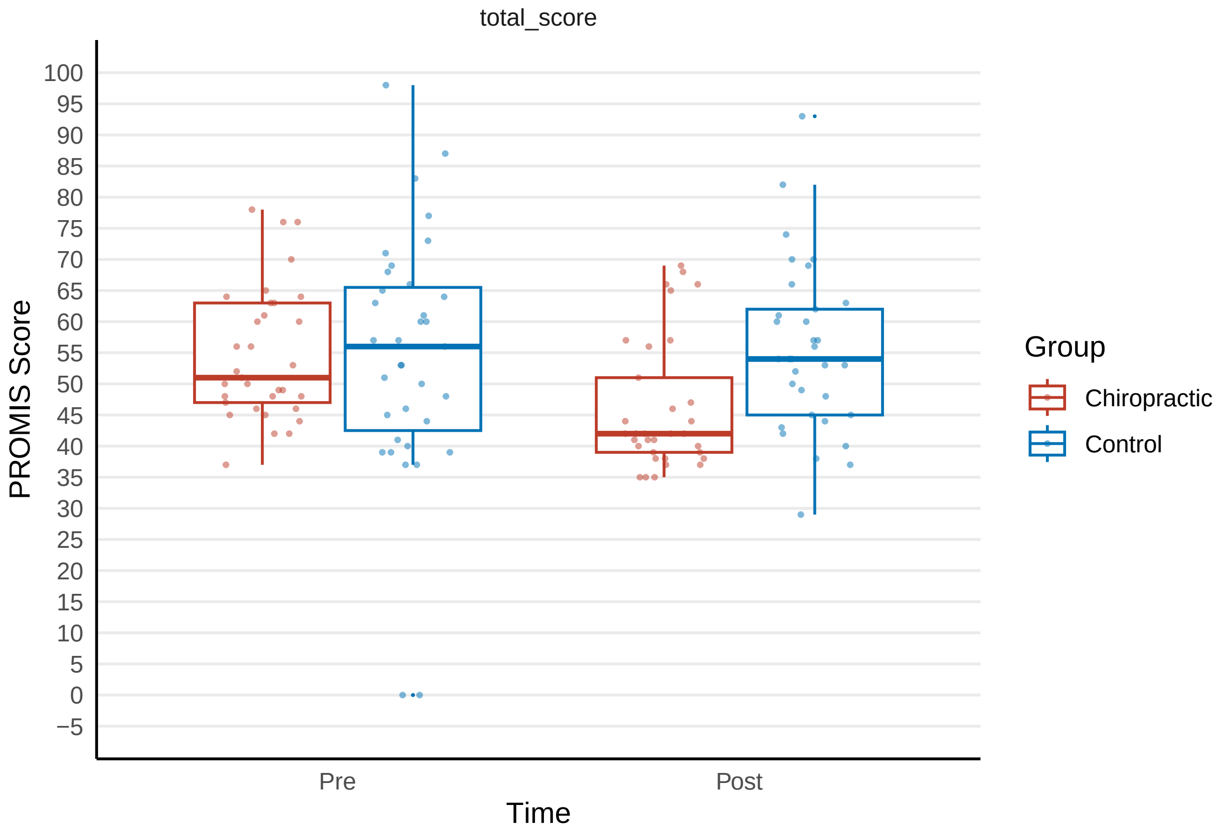
total_score
PROMIS Score
100
95
90
85
80
75
70
65
60
55
50
45
40
35
30
25
20
15
10
5
0
−5
Pre
Post
Time
Group
Chiropractic
Control

2 Statistical Comparison of Groups

2.1 PROMIS

2.1.1 Model

```
mdl.promis <- lmer(Value ~ Value.pre + Group*Component +
                       (1|PartId), subset(promis.table, Component != "total_score"))
```

2.1.2 Diagnostics

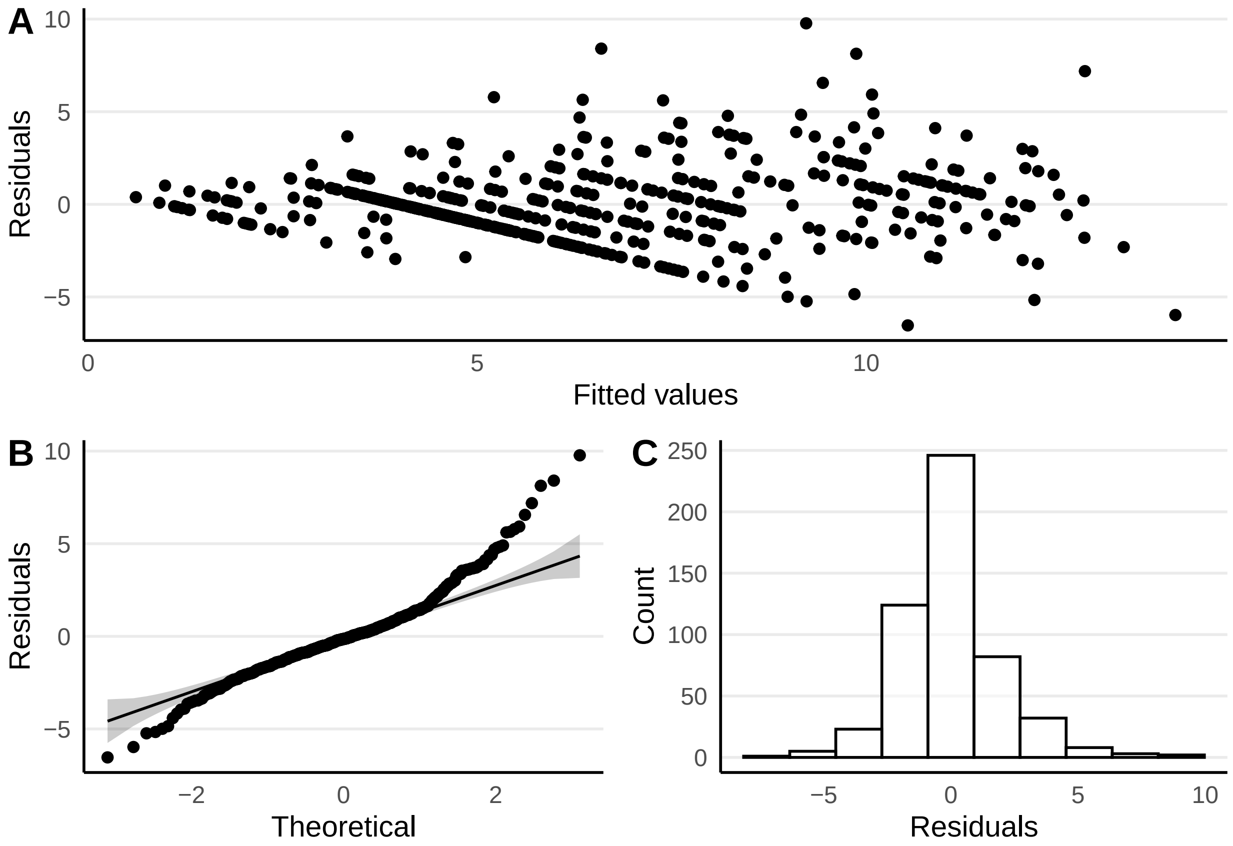

2.1.3 Analysis of Deviance

```
Analysis of Deviance Table (Type II Wald chisquare tests)

Response: Value
                   Chisq Df Pr(>Chisq)
Value.pre         75.405  1   < 2e-16 ***
Group             10.772  1   0.00103 **
Component        166.773  7   < 2e-16 ***
Group:Component   11.491  7   0.11860
---
Signif. codes:  0 '***' 0.001 '**' 0.01 '*' 0.05 '.' 0.1 ' ' 1
```

2.1.4 Variation across Componenets

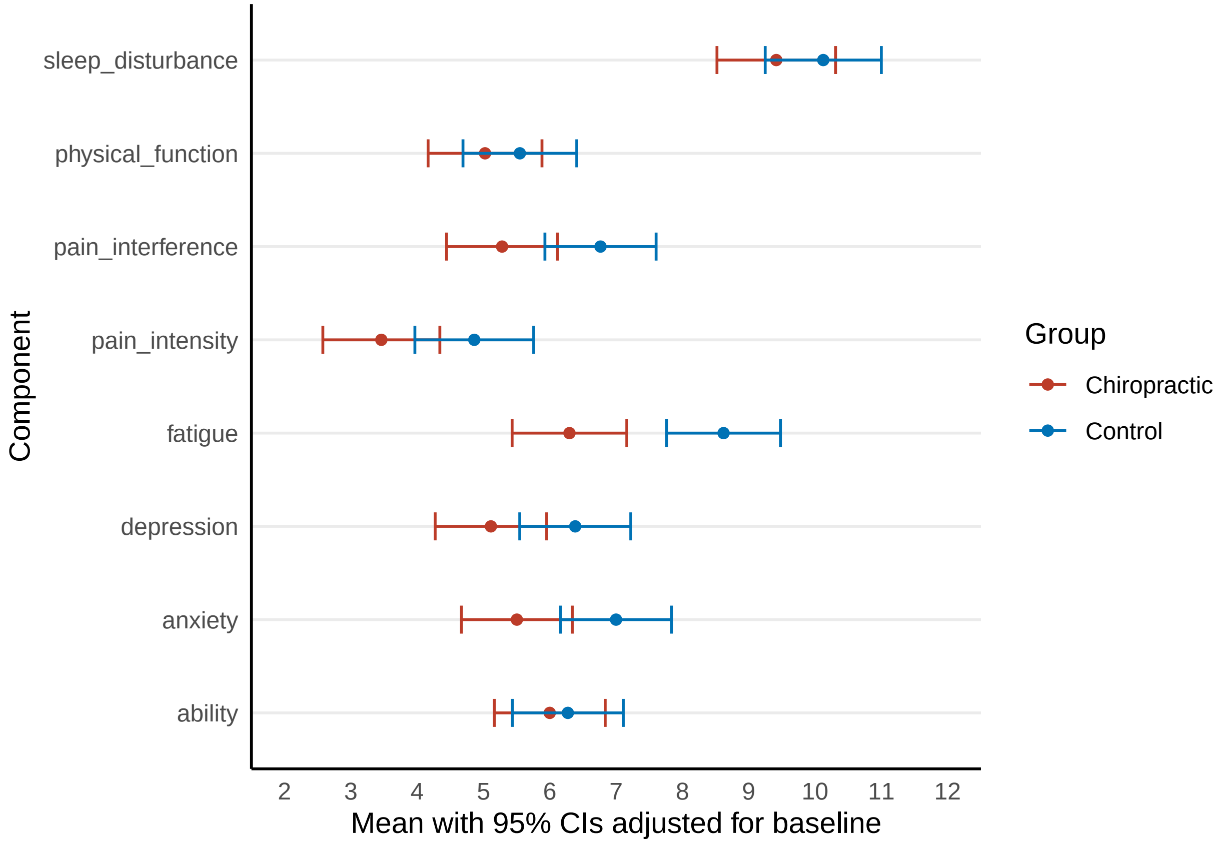

2.1.5 Between Group Statistics

Contrast	Component	Difference±SE [95% CI]	t[df], p-value
Chiropractic - Control	ability	-0.3±0.6 [-1.5, 0.9]	t[337.6]=-0.454, 0.65
Chiropractic - Control	anxiety	-1.5±0.6 [-2.7, -0.3]	t[337.8]=-2.489, 0.013
Chiropractic - Control	depression	-1.3±0.6 [-2.5, -0.1]	t[337.8]=-2.116, 0.035
Chiropractic - Control	fatigue	-2.3±0.6 [-3.5, -1.1]	t[337.7]=-3.867, <0.001
Chiropractic - Control	pain_intensity	-1.4±0.6 [-2.6, -0.2]	t[346]=-2.303, 0.022
Chiropractic - Control	pain_interference	-1.5±0.6 [-2.7, -0.3]	t[337.7]=-2.471, 0.014
Chiropractic - Control	physical_function	-0.5±0.6 [-1.7, 0.7]	t[337.6]=-0.874, 0.383
Chiropractic - Control	sleep_disturbance	-0.7±0.6 [-1.9, 0.5]	t[338]=-1.178, 0.24

2.1.6 Within Group Statistics

Group	Component	Estimate±SE [95% CI]	t[df], p-value
Chiropractic	ability	6±0.4 [5.2, 6.8]	t[337.9]=14.11, <0.001
Control	ability	6.3±0.4 [5.4, 7.1]	t[337.9]=14.751, <0.001
Chiropractic	anxiety	5.5±0.4 [4.7, 6.3]	t[337.7]=12.951, <0.001
Control	anxiety	7±0.4 [6.2, 7.8]	t[337.7]=16.47, <0.001

Group	Component	Estimate±SE [95% CI]	t[df], p-value
Chiropractic	depression	5.1±0.4 [4.3, 6]	t[339.5]=11.956, <0.001
Control	depression	6.4±0.4 [5.5, 7.2]	t[338.2]=14.997, <0.001
Chiropractic	fatigue	6.3±0.4 [5.4, 7.2]	t[347.7]=14.322, <0.001
Control	fatigue	8.6±0.4 [7.8, 9.5]	t[345.6]=19.75, <0.001
Chiropractic	pain_intensity	3.5±0.4 [2.6, 4.3]	t[353.4]=7.71, <0.001
Control	pain_intensity	4.9±0.5 [4, 5.8]	t[358.9]=10.673, <0.001
Chiropractic	pain_interference	5.3±0.4 [4.4, 6.1]	t[338.1]=12.41, <0.001
Control	pain_interference	6.8±0.4 [5.9, 7.6]	t[338.6]=15.872, <0.001
Chiropractic	physical_function	5±0.4 [4.2, 5.9]	t[345.7]=11.504, <0.001
Control	physical_function	5.5±0.4 [4.7, 6.4]	t[345.3]=12.723, <0.001
Chiropractic	sleep_disturbance	9.4±0.5 [8.5, 10.3]	t[357.1]=20.7, <0.001
Control	sleep_disturbance	10.1±0.4 [9.2, 11]	t[351.2]=22.74, <0.001

2.2 PROMIS (Total)

2.2.1 Model

```
mdl.promis.t <- lm(Value ~ Value.pre + Group,
                   subset(promis.table, Component == "total_score"))
```

2.2.2 Diagnostics

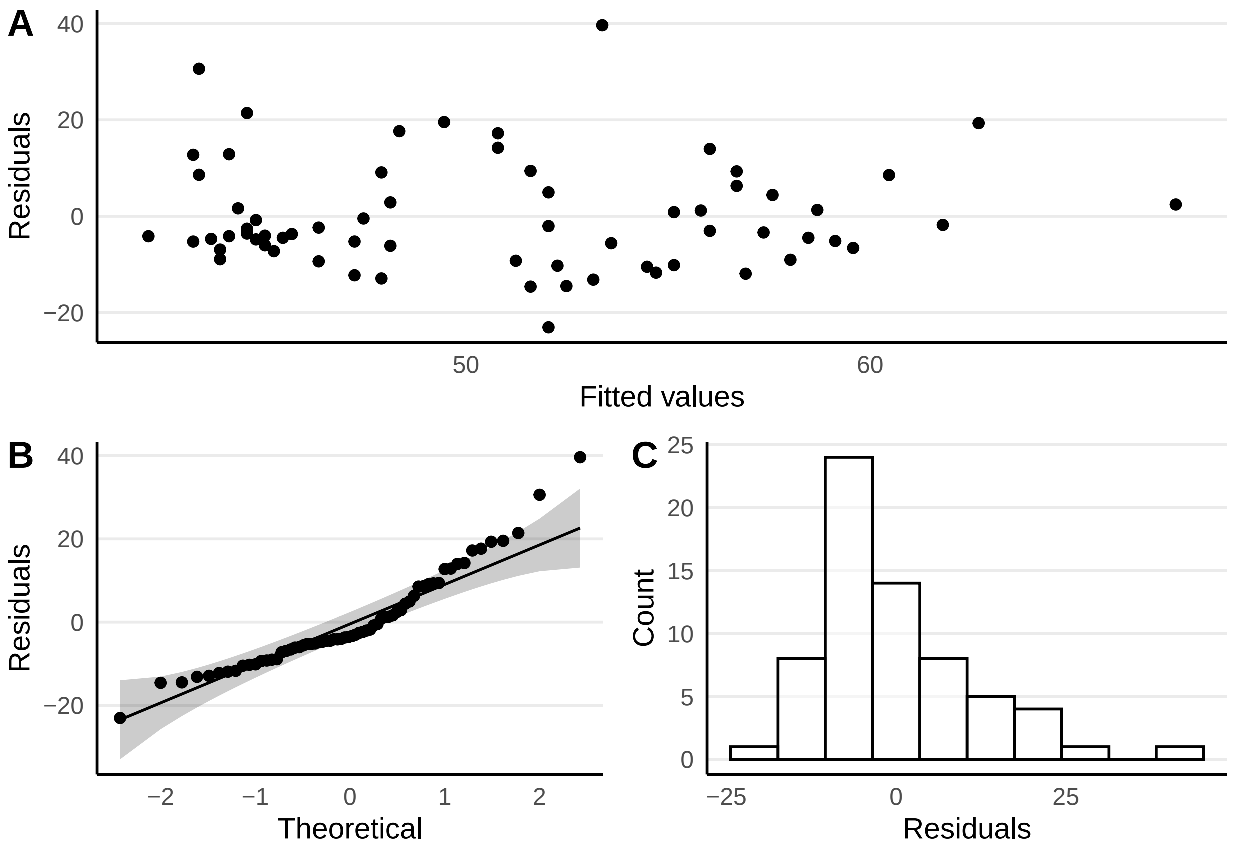

2.2.3 Analysis of Deviance

```
Anova Table (Type II tests)

Response: Value
           Sum Sq Df F value   Pr(>F)
Value.pre   902.3  1  6.7112 0.011890 *
Group      1474.8  1 10.9696 0.001537 **
Residuals 8469.8 63
---
Signif. codes:  0 '***' 0.001 '**' 0.01 '*' 0.05 '.' 0.1 ' ' 1
```

2.2.4 Between Group Statistics

Contrast	Difference±SE [95% CI]	t[df], p-value
Chiropractic - Control	-9±3 [-15, -4]	t[63]=-3.312, 0.002

2.2.5 Within Group Statistics

Group	Estimate±SE [95% CI]	t[df], p-value
Chiropractic	46±2 [42, 50]	t[63]=22.805, <0.001
Control	55±2 [51, 60]	t[63]=27.489, <0.001
